# Probiotics in Food Systems: Significance and Emerging Strategies Towards Improved Viability and Delivery of Enhanced Beneficial Value

**DOI:** 10.3390/nu11071591

**Published:** 2019-07-13

**Authors:** Antonia Terpou, Aikaterini Papadaki, Iliada K. Lappa, Vasiliki Kachrimanidou, Loulouda A. Bosnea, Nikolaos Kopsahelis

**Affiliations:** 1 Food Biotechnology Group, Department of Chemistry, University of Patras, GR-26500 Patras, Greece; 2 Department of Food Science and Technology, Ionian University, Argostoli, 28100 Kefalonia, Greece; 3 Hellenic Agricultural Organization DEMETER, Institute of Technology of Agricultural Products, Dairy Department, Katsikas, 45221 Ioannina, Greece

**Keywords:** probiotics, prebiotics, enhanced cell viability, encapsulation, synbiotics, functional food products

## Abstract

Preserving the efficacy of probiotic bacteria exhibits paramount challenges that need to be addressed during the development of functional food products. Several factors have been claimed to be responsible for reducing the viability of probiotics including matrix acidity, level of oxygen in products, presence of other lactic acid bacteria, and sensitivity to metabolites produced by other competing bacteria. Several approaches are undertaken to improve and sustain microbial cell viability, like strain selection, immobilization technologies, synbiotics development etc. Among them, cell immobilization in various carriers, including composite carrier matrix systems has recently attracted interest targeting to protect probiotics from different types of environmental stress (e.g., pH and heat treatments). Likewise, to successfully deliver the probiotics in the large intestine, cells must survive food processing and storage, and withstand the stress conditions encountered in the upper gastrointestinal tract. Hence, the appropriate selection of probiotics and their effective delivery remains a technological challenge with special focus on sustaining the viability of the probiotic culture in the formulated product. Development of synbiotic combinations exhibits another approach of functional food to stimulate the growth of probiotics. The aim of the current review is to summarize the strategies and the novel techniques adopted to enhance the viability of probiotics.

## 1. Introduction

Agricultural and food industry are constantly evolving entailing innovation phenomena that generate constant research and emerging technologies. The change of consumers’ preferences, needs and acceptances is a dynamic process, hence the maintenance of food quality via technology innovation is evident [1]. The cultural heritage of consumers, habits, and even sustainability factors can similarly affect technology innovations applied in the food industry [2,3].

Nowadays, consumers are becoming more health conscious and concerned about the beneficial value of food, thus directing manufacturers to emphasize on the promotion of functional foods. Therefore, the key for successful marketing and acceptance of novel foods depends not only on the concept of food quality throughout the chain but also to added value food functionalities [4]. These novel functional food products are either natural or processed foods that have been fortified with active compounds of known biological activity. These compounds when administered in defined quantitative and qualitative amounts, will provide clinically proven health benefits apart from those delivered by fundamental nutrients [5]. The development of probiotic food formulations is a key research area for the future functional food market. Economic forecasts expect an increase from 3.3 to 7 US$ billion for the global market of probiotic dietary supplements from 2015 to 2025 [6].

Probiotics are defined as “*live microorganisms which when administered in adequate amounts confer a health benefit on the host”* [7]. The health benefit of the hosts targets primarily on the modulation of gut microbiota [8,9]. Human gut microbiota includes the indigenous intestinal microflora that participates in diversified functions that improve host health [10]. Guidelines and information from the Food and Agriculture Organization of the United Nations (FAO)/World Health Organization (WHO) demonstrate the necessity for the probiotic strains to remain intact through the upper intestinal tract to ensure health promoting effects upon entering their site of action, regardless the delivery mode applied. For instance, to assure that, it has been stated that the so called “minimum therapeutic” level of viable probiotic microorganisms should be at least 10^6^ CFU/g of viable cells throughout the product shelf-life [11]. One of the most significant features of probiotics is the production of substances like antibiotics, anticarcinogens, or other compounds with health promoting or pharmaceutical properties [12,13,14]. A recently emerging concept attracting substantial research and industry interest with respect to probiotics is their role in the gut-brain axis. Recent evidence and ongoing studies suggest that intestinal microbiota has a bi-directional effect on mood disorders. Research is focusing on interactions among probiotic modulation of gastrointestinal tract (GIT) and neurological or neuropsychiatric disorders via the enteric and central nervous system [15,16,17,18]. The central nervous systems, the autonomic nervous system (sympathetic and parasympathetic arms), the enteric nervous system, the hypothalamic pituitary adrenal axis and the gut microbiota, form the brain gut microbiota axis [19]. Sharkar et al. [20] used the term psychobiotics to define probiotic bacteria than upon ingestion convey mental health benefits, also including prebiotics that will stimulate the activity of beneficial microflora [20].

Based on the amount of ingested food along with the effect of storage on probiotic viability, it was suggested that a daily intake of 10^8^–10^9^ cfu/g probiotic bacteria could survive the upper ingestion to exert their positive physiological functions in the human body [21]. Karimi et al. [22] have stated that ~100 g/day of probiotic products should be consumed in order to deliver about 10^9^ viable cells into the intestine on a regular daily basis [22]. Regular probiotics consumption was evidenced by dose-response studies, indicating the transient ability of *L*. *rhamnosus* GG to colonize GIT [23]. For instance, fifteen days after terminating the intervention in adults, *Lactobacillus* GG was recovered from stool samples of only 27% of the volunteers [23].

The inclusion of probiotics into a food matrix poses several technological challenges that need to be addressed [24]. These bacteria encounter a variety of stress factors, including temperature, acid and bile, increased concentration of certain ions or nutrient depletion, exposure to osmotic and oxidative stress in product matrices along with passage through the gastrointestinal transit that may detrimentally affect their viability and their functionality. Probiotics need either to adapt to such a dynamic environment or to be protected in order to survive and become available in adequate quantities and deliver their health benefits. Moreover, the addition of probiotics may alter the taste and aroma of the final food product due to production of different metabolites such as organic acids during fermentation and extended storage. Thus, it is essential, that the incorporation of a probiotic culture does not affect adversely the sensory and other quality characteristics of the product [25,26].

## 2. Probiotics and Delivery Systems

Food products supplemented with probiotics can carry a single or many different bacterial strains. Specifically, in fermented food products, probiotics can be added as starter cultures while other bacterial or yeast strains co-exist. However, the crucial point is for probiotics to be maintained in high counts during products shelf life. Although several bacteria strains and yeasts have been so far characterized as potentially probiotic, *Lactobacillus* and *Bifidobacterium* species constitute the main representatives thus being the ones most commonly studied. Both genera have a long history of safe use and have been characterized as “generally recognized as safe” (GRAS), being also dominant inhabitants in the microbiota of the human intestine [27]. Other species belonging to the genera of *Lactococcus*, *Enterococcus*, *Propionibacteria*, and *Saccharomyces* (e.g., *S. cerevisiae* and *S. boulardii*) are also included in the list of probiotics mainly because of their known health-promoting effects [28,29,30,31]. Bifidobacteria are anaerobic bacteria and most strains do not grow under atmospheric conditions of less than 90% air and 10% CO_2_. The optimal growth temperature is around 37 °C at a pH value of 6.5–7 [32]. For lactobacilli, the optimal growth temperature ranges from 30 to 40 °C, at a pH of 6.5. Also, some thermophilic strains grow well and present highly activated metabolism at temperatures around 45 °C [33,34,35]. Recent research is focusing on the characterization of novel strains with beneficial impact that constitute natural inhabitants in the gut. For instance, the probiotic and beneficial potential of *Akkermansia municiphila* with respect to immuno-modulation and gut modulation has been investigated [36]. *Faecalibacterium prausnitzii* strains, belonging to Firmicutes phyla, were recently isolated and evaluated for their probiotic potential [37]. The specific bacterium has been reported to decrease in patients with intestinal disorders, including Crohn’s disease, whereby it was proposed that application of *F*. *prausnitzii* as a probiotic could modulate dysbiosis phenomena in the gut [38].

**Figure 1:**
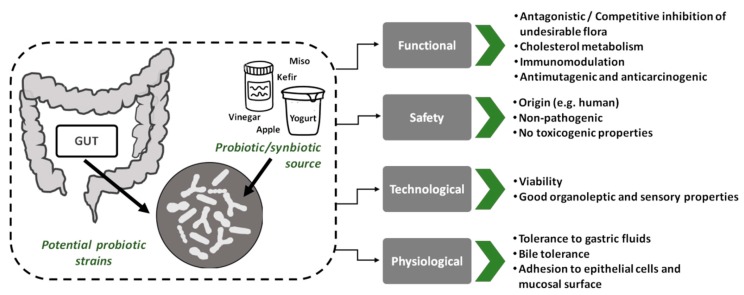
Criteria for the selection of probiotic strains.

Several systems have been developed for the delivery of probiotics to the GIT such as pharmaceutical formulations and food-based products. Pharmaceutical preparations in particular are considered more effective compared to commercial food-based carrier systems although their preference mainly depends on consumers’ perception [4]. Examples of pharmaceuticals for the delivery of probiotics currently include, capsules, liquids, powder beads, and tablets [39]. Strain selection and differentiation is considered a crucial step in both pharmaceutical and food systems (Figure 1). Likewise, an issue that needs evaluation is the fact that the preparation methods for the delivery matrix may affect cell viability and effective target release in the GIT.

Food-based probiotic products account for a large number of probiotic formulations and can be divided in two distinct categories: Dairy products e.g., cheeses, yogurts, ice cream, milk, acidified milks and creams and non-dairy products, e.g., meats and meat products, bread or other fiber snacks, chocolates, fruit juices and other fruit preparations [40,41,42,43,44]. The vast availability of food products makes them a good and potentially effective carrier system for the delivery of probiotics. Nevertheless, their ability to deliver viable cells to the human intestine may differ considerably, depending largely on the physicochemical properties of each composite food matrix.

## 3. Significance of Cell Viability

Bacterial viability refers to the ability of a cell to grow and subsequently generate a colony of cells under defined environmental conditions [24]. Viability is generally considered as a prerequisite for the functionality of probiotics as it relates with consumers health-promoting properties, thus it constitutes an industrial challenge. Several studies have reported the significance of viable cells with respect to functional properties on probiotic characteristics. Antimicrobial compounds and short chain fatty acids are indicative metabolites produced from viable colonies [45]. Galdeano et al. conducted a study in mice on the effect of viable and non-viable lactobacilli and their persistence in gut and mucosal immune stimulation [46]. The authors demonstrated that the viability of bacteria was necessary to stimulate the gut immune system. In another clinical study, Pelletier et al. suggested that viable cells are more effective in lactose digestion compared to non-viable probiotic cells [47]. On the other hand, certain reports have reported functionalities associated with both alive and non-viable probiotics [48,49].

As a result, scientific research has recently focused on many different innovative techniques targeting to enhance the viability of probiotic cells during products’ shelf-life [50]. Many of these methods have been proved to be quite successful, nevertheless enhanced shelf-life of probiotics does not necessarily provide the appropriate robustness of a culture when exposed to the challenging conditions of the GIT [39]. Each probiotic strain may exhibit a different response on various stress factors of the digestive tract. Cell membrane may be affected after consumption, by several factors such as the gastric acid, bile salts, various digestive enzymes, the food matrix or even the host’s microbiota [51]. Likewise, probiotic strains can be detected within the gastrointestinal tract as viable, dormant, active or dead depending on the environmental conditions and the capacity of the strain to survive [51,52]. On the other hand, certain studies have demonstrated that viability is not mandatory for all probiotic effects as not all cell mechanisms are directly related to viability since even the dead cells proved to provide beneficial effects to the consumer [53]. Research should focus more on the interactions occurring in the gut after the consumption of probiotics considering the indigenous gut microbial diversity of each human [54]. Hence, the main challenge would be to construct a model that could provide a more personalized diet based on ingredients that can reassure consumers optimal health [55]. Implementation of metabolomics as part of transcriptomics and proteomics could elucidate the understanding of interactions between the metabolic pathways of gut microbiota and the host along with the effect of age, gender, lifestyle, diet to allow gut microbiota modulation. For instance, genome-scale metabolic modeling could elucidate host-microbiome-diet interactions to study the microbial gut metabolism in physiological and dysbiosis states [56].

## 4. Factors Affecting Viability of Probiotics

**Figure 2:**
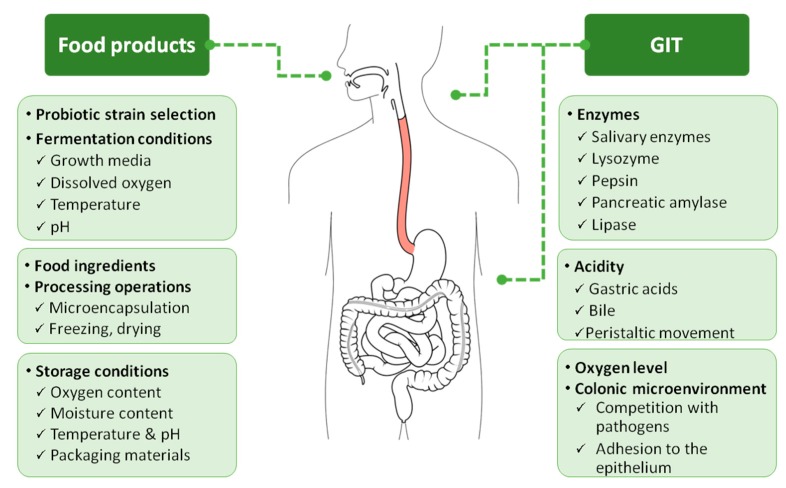
Factors affecting viability of probiotics in food products (during processing and storage), as well as in the gastrointestinal tract (GIT).

Many factors have been identified to influence the viability of probiotics in food products during processing and storage (Figure 2). These factors include intrinsic parameters of the product like pH, titratable acidity, oxygen, water activity, presence of salt, sugar and other compounds (hydrogen peroxide, bacteriocins, artificial flavoring and coloring agents etc.), processing parameters including fermentation conditions (incubation temperature, heat treatment, cooling and storage conditions of the product, packaging materials, scale of production), and finally microbiological parameters (strain of probiotics employed, rate and proportion of inoculation) [39,52].

### 4.1. Chemical Factors

Food components such as additives (sugars, salt, antimicrobials, aroma compounds, or even bacteriocins) can positively or negatively affect probiotic cells’ viability. Antimicrobial compounds and bacteriocins can significantly challenge the viability of probiotics in a food matrix, especially during storage, whereas prebiotics are known to have a positive effect on the viability of probiotics [57,58].

Oxygen levels and redox potential are also among the important factors affecting the viability of probiotic cultures, especially during storage, and primarily refer to anaerobic bacteria such as bifidobacteria [59]. The effect of oxygen on cell viability largely differentiates within the genera encountered in gut microbiota. For instance, lactobacilli are more tolerant to oxygen than bifidobacteria, to the point where oxygen levels are rarely an important issue in maintaining the survival of the former [60]. Therefore, oxygen concentration and oxygen permeability of the packaging should be maintained at low levels to effectively control losses in probiotic viability. Several methods have been proposed to reduce the oxygen content in packaged probiotic foods, e.g., vacuum packaging, addition of antioxidants or oxygen scavengers like ascorbic acid [61]. The sensitivity of oxygen, including bifidobacteria that are obligate anaerobes, limits their survival and use in industrial applications [62,63]. Several methods have been used to decrease oxygen levels during fermentation of products, like fermentation under vacuum-anaerobic conditions, addition of oxygen scavengers or genetic manipulation of bifidobacteria [64].

The water activity is another factor that may affect probiotic survival. Cell viability is particularly affected when a dry food matrix has an elevated water activity (*a_w_* > 0.25) [65]. Titratable acidity and low pH values can also affect the viability of probiotics during storage. Usually, fruit juices with low pH and a high organic acid content impose a significant stress challenge to probiotics. Champagne, et al. [66] inoculated *Lactobacillus rhamnosus* R0011 in an apple-pear-raspberry juice blend, at 4.5 × 10^9^ CFU/250 mL portion, and the viability was monitored during storage under conditions simulating consumer handling [66]. When the juices remained closed in the PET (polyethylene terephthalate) bottles, the viable population gradually dropped by 75% over 5 weeks of storage at 7 °C, while in opened bottles the reduction was only between 20% and 40%. Moreover, Ding and Shah [67] investigated the survival of eight different strains of free and microencapsulated probiotic bacteria in orange and apple juices during six weeks storage [67]. They reported that encapsulated probiotic bacteria survived in fruit juices throughout the entire storage period, whereas free probiotic bacteria showed a reduction in viability within five weeks. In general, fruit juices containing encapsulated probiotic bacteria were more stable than those containing free probiotic microorganisms. More recently, the survival of free *Bifidobacterium longum* NCIMB 8809 cells was studied during refrigerated storage for 6 weeks in model solutions [68]. Among all factors affecting cell viability, the most important ones that negatively changed cells viability were pH (3.2–4.0), citric acid (2–15 g/L), protein (0–10 g/L), and dietary fiber (0–8 g/L), with the pH and citric acid being the most significant. The highest cell survival (less than 0.4 log decrease) after 6 weeks of storage at 4 °C was observed in orange and pineapple, both of which had a pH value of about 3.8. Although the pH value of grapefruit and blackcurrant were similar (pH ∼ 3.2), there was a 0.2 log reduction difference deriving probably due to the high amount of citric acid of grapefruit (15.3 g/L). The large decrease in cell viability (∼8 logs) noted in pomegranate and strawberry juices was attributed to the high levels of phenolic compounds and the very low pH of these juices (pH ≤ 3) [68]. Also, large differences in acid resistance of free lactobacilli and bifidobacteria cells have been observed when added to orange, pineapple and cranberry juices and further stored at 4 °C [69]. Overall, encapsulation techniques are well documented to convey increased tolerance of probiotics in low pH and high water activity conditions, such as those encountered in fruit juices.

### 4.2. Biological Factors

Many biological factors such as strain type, antagonism with starter cultures, product natural microflora, produced enzymes, post-acidification and occurrence of various pathogenic or spoilage microorganisms are known to influence the viability of probiotics [70,71]. A desirable criterion for the selection of probiotic is to present functional, technological and safety properties, without providing negative features. The selected probiotic strain may show antagonistic effects against various microorganisms resulting in losses of cell viability. In many cases, the added probiotic culture may be affected by the starter culture used for food fermentation. On the other hand, the appropriate probiotic strain can demonstrate several antagonistic mechanisms including competition for nutrients, coaggregation with pathogens, and immune system stimulation [71]. A special reference needs to be addressed regarding the manufacture of fermented probiotic meat products as they are more complicated than other probiotics-containing products. The reasons associate with raw material characteristics [72] such as high salt content, low pH and water activity due to the acidification and drying processes. Fermented fish products have been also studied as a method of probiotic delivery [73]. In general, cell viability in a fermented meat environment will most likely be strain-dependent [72]. Therefore, the choice of appropriate microorganisms to be applied as probiotic in a fermented meat matrix is considered to be critical [74,75].

### 4.3. Physical Factors

Physical factors that affect probiotic survival include storage temperature, drying conditions or oxygen levels. Cell membranes of probiotics get damaged during freezing process due to mechanical stresses imposed by the development of ice crystals in the external medium (intercellular space) or inside the cells. The size of ice crystals can be reduced by applying rapid freezing rates that result in smaller ice crystals [76,77]. On top of that, the survival of probiotics may be further reduced during thawing where the cells are exposed to osmotic stresses [78].

The fermentation temperature also affects the viability of probiotic microorganisms; with the optimum temperature range for growth for the majority of LAB being within a range of 30–43 °C. Some bacteria, however, such as those of yogurt cultures and *L. acidophilus* can grow at 45 °C. Usually, temperatures above 45 °C during processing could negatively affect probiotic survival [60]. Species of bifidobacteria isolated from the human intestinal tract such as *B. longum* subsp. *infantis*, *B. breve*, *B. bifidum*, and *B. adolescentis* show an optimum growth temperatures in the range of 36–38 °C, whereas *B. animalis subsp. lactis* can grow at higher temperatures of 41–43 °C [59].

In order to maintain cultures for experimental and industrial uses, drying can be employed to reduce the cost of frozen storage and transportation; i.e., probiotic foods are sometimes dried in order to increase their shelf life at ambient temperature and to reduce the cost of frozen storage at very low temperatures, −20 to −40 °C [79,80]. Several drying methods can be applied however, freeze-drying, spray-drying and vacuum-drying [79,81,82] are the most applicable methods for bacterial culture preservation.

Spray-drying is an economical and flexible method for drying liquid foods; however, its application to preserve probiotic cultures usually entails significant losses in cell viability due to high temperatures, dehydration and osmotic phenomena. Based on several studies, the survival of probiotic cultures during spray-drying depends on several factors such as the species and strain of probiotics used, the drying parameters (outlet air temperature, type of atomization), and the drying and growth medium [60,83]. Freeze-drying, on the other hand, is an expensive process that maintains largely the viability of the probiotic cells [82,84,85].

## 5. Strategies for Enhanced Probiotic Viability

### 5.1. Selection of Probiotics

The selection and characterization of new species (e.g., *Lactobacillus, Bifidobacterium, Propionibacterium, Faecalibacterium*) constitutes a major field of research with respect to the selection of probiotics. The criteria for a strain to be characterized as a probiotic either for food or nutraceutical applications are constantly evolving and developing. These criteria can be roughly divided in four distinct categories: 1. technological, 2. safety, 3. functional and 4. physiological characteristics [60,86,87,88]. Viability during food processing and/or storage and survival after the passage in the upper intestinal transit along with the ability to exert health benefits to the host constitute the most important criteria for the probiotic selection. Most bacteria are unable to survive against the harmful environment during the upper GIT transit, including that of the gastric and duodenum. Hence, selection of the appropriate probiotic strain is a key factor in formulating products with viable probiotic cultures.

Several studies showed losses of 6 to 8 log cfu/g of probiotic bacteria during artificial gastric digestion [89,90,91], implying that the residual probiotic counts are not sufficient to exert health beneficial effects. Vijayakumar, et al. [92] studied the probiotic potential of the lactic acid bacteria *L. plantarum* KCC-24, that was isolated and characterized from Italian ryegrass (*Loliummulti florum*) forage [92]. The isolated strain exhibited significant antifungal activity against several strains, it was susceptible to numerous antibiotics, survived in low pH, was resistant to simulated gastric juices and bile salts (0.3% *w*/*v*). Also, *L. plantarum* KCC-24 exhibited good proteolytic activity, potent antioxidant and hydrogen peroxide resistance properties, thus proving to be an excellent probiotic candidate.

Turková, et al. [93] have evaluated eleven strains of *Lactobacillus* included in the Culture Collection of Dairy Microorganisms (CCDM) for selected probiotic properties such as survival in the gastrointestinal fluids, antimicrobial activity, and competition with non-toxigenic *Escherichia coli* O157:H7 for adhesion on Caco-2 cells [93]. All strains presented significant antimicrobial activity, whereby the three best performing strains inhibited the growth of at least sixteen indicator pathogenic strains. The degree of competitive inhibition of non-toxigenic *E. coli* O157:H7 adhesion on the surface of Caco-2 cells was found to be strain-dependent. However, only three of the strains were selected for additional studies of antimicrobial activity, i.e., *L. gasseri* CCDM 215, *L. acidophilus* CCDM 149, and *L. helveticus* CCDM 82 [93].

The effect of carrier food matrix on in vitro gastrointestinal survival and adhesion ability of probiotic *L. acidophilus* LA-5, *B. animalis subsp*. *lactis* BB-12 and *P. jensenii* 702 were evaluated on dairy products [94]. The carrier food matrix had a significant influence on the in vitro gastrointestinal tolerance of all probiotics upon exposure to low pH (pH 2.0) and 0.3% bile. For instance, Terpou et al. [95,96], reported a significant tolerance improvement in the case of ice cream and a moderate one in the case of yogurts, while Ranadheera et al. [94] observed that in vitro adhesion ability of probiotics was influenced by the carrier food matrix, with fruit yogurt providing the most favorable results [94].

Following an exposure to acidic conditions, *B. longum* presented an enhanced survival when compared to *B. infantis*, *B. adolescentis* and *B. bifidum* [97]. Amongst various strains of lactobacilli investigated *L. acidophilus* ATCC 4962, *L. casei* ASCC 290 and *L. casei* ASCC 292 were the most acid-tolerant. Live cell density was >10^7^ cfu/mL after 2 h incubation at pH 2.0, whereas *L. casei* ASCC 1520, *L. casei* ASCC 1521, *L. casei* ASCC 279, *L. casei* ATCC 15820 and *L. casei* CSCC 2607 were the most acid-sensitive strains with only 104 total cfu/mL remaining after 2 h incubation [98]. Lo Curto, et al. [99] investigated the survival of three commercial probiotic strains (*L. casei subsp. shirota*, *L. casei subsp. immunitas*, *L. acidophilus subsp. johnsonii*) in the human upper GIT using a dynamic gastric model (DGM) of digestion followed by incubation under duodenal conditions. Higher survival was observed in stationary phase for all strains [99]. The *L. acidophilus subsp. johnsonii* exhibited the highest survival rate in both water and milk. Hence, it is well documented that the ability of the probiotic strain to tolerate gastric acid conditions and the toxicity of bile salts is one of the key factors to be fulfilled in the probiotic selection criteria.

### 5.2. Strain Adaptation on Food Matrix and Human Microenvironment

Traditionally, probiotic strains are selected based on stress-resistance phenotypes that guarantee their survival through the GIT and their survival rates within each food matrix. Production, storage, and use of LAB impose environmental stress on bacterial cells. Especially during industrial fermentation, LAB encounter a number of stress conditions such as low temperature, low pH, and low water activity [100]. Although the application of physical stress to microorganisms, is the most widely used method to induce cell inactivation and enhance food stability, microorganisms have evolved both physiological and genetic mechanisms to tolerate some extreme conditions in order to survive [71]. This is clearly of major significance to the food industry in relation to antagonism against pathogens or spoilage organisms [101]. Interestingly, the application of different stress conditions to improve the viability and stability of probiotics is of significant interest.

Probiotics sustain several molecular mechanisms to respond the environmental stress encountered either during processing or during ingestion and passage in the GIT. Hence, by elucidating the underlying mechanisms probiotics with high and enhanced viability could be developed. Stress adaptation is one of the strategies to improve the survival of probiotics. This is achieved by pre-treating (preculturing) the bacterial cells at a sublethal stress condition prior to exposure to a more harsh or lethal environment [102]. This approach allows probiotic bacteria to develop adaptive stress responses leading to an increase in their survival compared to those that are directly shifted into the same lethal stress condition [103]. Adaptive responses towards various types of stress, i.e., heat, cold, acid, bile salts, osmotic, oxygen, high pressure and nutrient starvation have been applied for the production of high tolerant probiotic strains [104,105]. These features usually resemble environmental conditions typically encountered by probiotics during human GIT transit, exposure to industrial-scale production protocols and in the food matrix environment [106]. Acid and osmotic stress, occurring from lactic acid production and application of food additives, are the predominant stress factors during yoghurt manufacture and refrigerated storage [107]. Recent advances in post-genomics technologies, i.e., transcriptomics and proteomics, have provided novel insights into the mechanisms by which probiotics counteract environmental stresses [108].

In their various applications in the food and feed industry, LAB can be exposed to osmotic stress when large quantities of salt or sugar are added in the product. Thus, they need to adapt to such a change in their environment in order to survive. Accumulation of compatible solutes (uptake or synthesis) under hyper-osmotic conditions and releasing (or degrading) them under hypo-osmotic conditions comprises one of the possible mechanistic actions. Compatible solutes may also stabilize enzymes and thereby provide protection not only against osmotic stress but also against high temperature, freeze thawing, and drying processing protocols [109,110,111]. Similarly, polyphenols present in the food matrix (e.g., blackcurrant juice) might equally impair or enhance lactobacilli growth depending on the concentration of anthocyanins [112].

Adaptive stress responses in probiotics are also associated with the alteration of various physiological features and structural cell components [113]. It has been reported that for acid tolerance response several mechanisms are linked to pH homeostasis by the proton-translocating F1F0-ATPase, alteration of cell membrane fatty acid composition that leads on the modification of cell membrane properties, the increase of alkalinity of cytoplasm by the activity of arginine deaminase, urease and glutamine decarboxylase and the production of several stress proteins [114]. Furthermore, the response to osmotic stress involves the accumulation of compatible solutes and activation of membrane associated proteins for maintaining turgor pressure of the cell [115].

Several reports have suggested that pre-adaptation can enhance the survival of probiotics in a food system [116,117]. Nevertheless, it is considered that adaptive responses are highly strain-dependent and vary largely according to the type of stress along with other experimental conditions [117]. Settachaimongkon, et al. [118] investigated the effect of preculturing *L. rhamnosus* GG and *B. animalis* subsp. *lactis* BB12 under sublethal stress conditions, on their survival and metabolite formation in set-yoghurt [118]. Both probiotic strains presented adaptive stress responses resulting in viability improvement without any adverse effects on milk acidification.

Osmotic stress is also considered a stress factor related with probiotic viability in food matrices. For example, flow cytometric studies, performed on some lactobacilli exposed to varying levels of sugar concentrations, showed losses of probiotic viability due to osmotic stress [119]. Gandhi and Shah [120] used flow cytometry to evaluate the probiotic cell viability when subjected to varying NaCl concentrations (0–5%) and studied the metabolic state of cells [120]. Double staining of the cells and metabolic activity measurements revealed the degree of cell injury when subjected to NaCl concentrations. They also reported on the variability in salt resistance of three probiotic strains; the salt resistance of *L. casei* was found to be higher than *L. acidophilus* and *B. longum.*

Oxygen stress is another hurdle that probiotic microorganisms may encounter during food production. Oxygen dissolves easily in milk and other liquid foods whereas oxygen permeation through the package could affect probiotic viability [121]. Some studies showed that gradual exposure to increasing concentrations of dissolved oxygen could improve viability in *B. longum, L. acidophilus* and *Bifidobacterium* sp. cultures [122,123].

Acid stress adaptation has been originally investigated for food pathogens since it can significantly influence the survival of these bacteria in acidic environments. Such a mechanism, although undesirable for pathogenic organisms, can be also effective in altering the survival of probiotics in acidic matrices and through their passage in the GIT. Several studies have focused on the potential stress response mechanisms of lactobacilli in order to improve their capacity to survive and function under industrial production conditions [124,125]. The effect of heat shock and induction of a stress response in *Lactobacillus* sp. and further exposure of *L. rhamnosus* GG cells to pressure pretreatment for improving thermo-tolerance upon exposure at 60 °C was also investigated [126,127,128]. Moreover, application of non-lethal heat shock allows bacteria to tolerate a second heat stress higher in intensity [129]. In another study, salt adaptation of *L. paracasei* NFBC 338 (0.3 M NaCl for 30 min) improved its viability during spray drying [126,127].

### 5.3. Selection of Food Packaging Systems

The physical properties of packaging material and packaging techniques could influence the survival of probiotics [130]. The majority of dairy probiotic and other products are stored and sold in the market in plastic packages with high oxygen permeability. Bifidobacteria, in particular, are anaerobes with a high susceptibility to oxygen, therefore a packaging with high oxygen permeability could affect their viability during storage. In this context, several factors such as temperature, relative humidity and crystallinity of the film matrix may affect the permeability of packaging material and thereby alter the probiotic viability [131,132]. Miller, et al. [133] have reported that packaging of yogurt in a container made of a material with good oxygen barrier properties along with an oxygen scavenging agent provides the most favorable environmental conditions for preserving probiotics [133]. Shah [121] reported greater survival of *L. acidophilus* in yoghurt packed in glass bottles rather than in plastic containers and suggested the use of packaging materials with greater thickness for better survival of both *L. acidophilus* and bifidobacteria in yoghurt [121]. Cruz, et al. [134] studied the viability of probiotics in yogurts packed in different plastic containers with different oxygen permeability along with the addition of glucose oxidase [134]. As expected, low oxygen permeability rates resulted in higher probiotic viability, even though a higher post-acidification and organic acid production was observed. Therefore, low oxygen permeability in containers favors the survival of probiotic cultures. However, the use of glass containers, having limited oxygen permeability, increases the material cost for probiotic packaging. Alternative packaging methods such as the inclusion of oxygen scavengers or absorbents, vacuum packaging or active packaging with the inclusion of oxygen barrier materials could provide a more cost effective solution [60].

### 5.4. Addition of Compounds as Probiotic Promoters

Different compounds may be added in probiotic products to act as growth promoters (e.g., sugars, vitamins, minerals, prebiotics) or as protectants against processing conditions (e.g., skim milk powder, whey protein, glycerol, lactose). Also, the use of prebiotics as protectant for probiotic microorganisms has been gaining considerable interest. The prebiotic definition was quite recently revised as “*a substrate that is selectively utilized by host microorganisms conferring a health benefit*” [135].

Tian, et al. [136] studied the feasibility of casein glycomacropeptide (GMP) hydrolysates as potential prebiotics in yogurt. The growth performance of *B. animalis* subsp. *lactis* (Bb12), *L. bulgaricus* and *S. thermophilus* in the presence of GMP hydrolysate produced with papain (GHP) was evaluated. The results showed an improvement in the growth of *S. thermophilus*, but lower effect on the growth of *L. bulgaricus*. However, the viable count of Bb12 of the yogurt obtained with the addition of 1.5% GHP was about four times higher than that of the control without GHP addition [136]. Shin, et al. [137] found that the viability of commercial *Bifidobacterium* spp. in skim milk improved by 55.7% after 4 weeks of refrigerated storage when fructo-oligosaccharides (FOS) were added [137]. The addition of oligofructose as prebiotic into yogurt (1.5% *w*/*v*) improved the viability of the probiotic organisms during refrigerated storage [138]. Also, inclusion of other protective compounds such as whey protein hydrolysate [139], inulin [140], and whey protein concentrate [141] was found to promote probiotic viability. Akalin, et al. [142] showed that incorporation of FOS in yogurt improved the viability of bifidobacteria [142].

Several of the aforementioned compounds (e.g., glycerol, skim milk powder, whey proteins and sugars) have been also utilized as thermo- or cryo-protectants during the production of probiotics [60]. Capela et al. [138] have reported that addition of glycerol prior to freeze drying reduces the osmotic pressure and therefore assists the adaptation of probiotics in the environmental conditions [138]. Skim milk solids have been reported to form a protective coating on cell wall proteins and stabilize the cell membrane [143]. Several carbohydrates also exert protective effects for probiotic bacteria during freeze drying. Trehalose is a well-known inert (non-reducing disaccharide) cryoprotectant due to its remarkably high glass transition temperature, and its ability to engage in strong ion–dipole interactions and hydrogen bonding with other biomolecules, enabling better survival of *L. acidophilus* [111].

Innovative delivery strategies based on incorporation of probiotic bacteria into protective matrices, such as whey proteins, proved the ability to provide extra protection for bacteria against environmental stresses. In a recent study Cordeiro et al. [144] evaluated skim milk, previously fermented by a *L. casei* or by *P. freudenreichii* strain, with the addition of whey protein isolate (WPI). Their results revealed that supplementation with 30% (*w*/*v*) of WPI increased the survival rate of both strains when challenged with acid, bile salts, heat and cold stress, compared to fermented skim milk without the addition of WPI [144]. Enhanced viability of freeze-dried *L. plantarum* cells using of whey protein microgels has also been presented. The protective effect was further improved with the increase of WPI concentration [145].

The influence of the carrier matrix should be also evaluated to select the appropriate probiotic strains for selective applications. The incorporation of cells in hydrocolloids has already demonstrated great potential in the view of bioactive solutions for functional food. Likewise, novel approaches using edible films and coatings have been shown to improve cell population survival [146,147,148,149]. In a recent work *L. rhamnosus* GG interplay survivability was studied, comprising selected biopolymers, in the presence of whey protein concentrate (WPC) as a potential vehicle [149]. The authors suggested kappa-carrageenan/locust bean gum and sodium alginate as the most promising performing system. Additionally, they concluded that the inclusion of WPI further increased *L. rhamnosus* GG stability. A successful example has also been presented by Pavli et al. [150] where Na-alginate edible films were evaluated as vehicles for delivering probiotic bacteria to sliced ham using high pressure processing (HPP). The probiotic-supplemented films were found to be efficient for probiotic delivery on the sliced ham, regardless the previous HPP treatment, since viability was fairly constant (>10^6^ CFU/g) throughout storage time regardless the storage temperature (4 °C, 8 °C, 12 °C) [150].

### 5.5. Encapsulation of Probiotics

**Figure 3:**
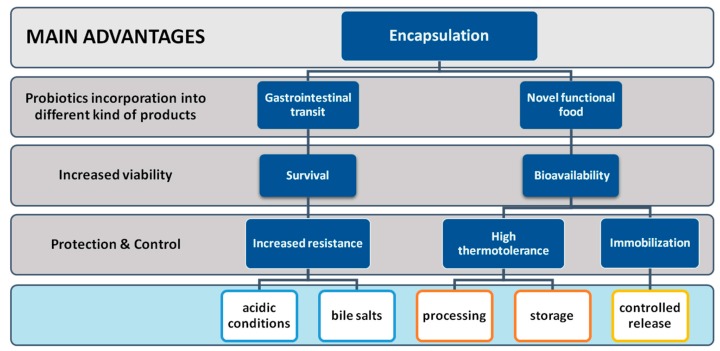
Schematic presentation of the main advantages of probiotics encapsulation.

Lately, the most investigated method for improvement of probiotic survival and delivery of bioactive compounds is encapsulation (Figure 3). Probiotics encapsulation is known to enhance stability, facilitate handling and storage of probiotic cultures and protect sensitive probiotic lactic acid bacteria from oxygen, freezing and acidic conditions during production, storage and gastrointestinal transit [151]. So far, several parameters have been identified to affect the efficacy of encapsulation in probiotic protection, the method and the wall materials used, the pH, the initial cell population, the probiotic strain, and the food matrix among others [148,152,153,154].

#### 5.5.1. Encapsulation Materials

Several materials have been applied for probiotic cells encapsulation, including polysaccharides (alginate, plant/microbial gums, chitosan, starch, k-carrageenan, cellulose acetate phthalate), as well as proteins (gelatin, milk proteins) and fats [155,156,157,158] (Table 1). Based on recent studies the use of gum or biopolymeric matrices can retain the viability of encapsulated probiotic cells compared to free ones, providing in many cases antimicrobial effects [159,160,161,162] against pathogenic or spoilage microorganisms. Casein-based microencapsulation proved to improve the viability of *Lactobacillus F19* and *Bifidobacterium Bb12* during freeze drying and subsequent storage [163]. Phoem, et al. [164] microencapsulated *B. longum* using extrusion and emulsion techniques for protection against sequential exposure to simulated gastric and intestinal juices, refrigeration storage and heat treatment. These results showed that encapsulated *B. longum* with *Eleutherine americana* and oligosaccharide extract prepared by the extrusion technique, survived better than that by the emulsion technique under adverse conditions. The viability of encapsulated cells was better than free cells at 65 °C for 15 min [164].

Encapsulation of bifidobacteria in poly-(vinylpyrrolidone)-poly-(vinylacetate-co-crotonic acid) (PVP:PVAc-CA) interpolymer complex microparticles under supercritical conditions was applied by Thantsha, et al. [165]. They reported that the produced microparticles had suitable characteristics for food applications and protected the bacteria in simulated gastrointestinal fluids as well as improved the shelf life for 12 weeks at 30 °C [165]. The strain *B. adolescentis* (ATCC 15703) was entrapped within microcapsules prepared using 10.00% (*w*/*w*) chickpea protein isolates cross-linked with 0.20% (*w*/*v*) of genipin, or in the presence of 0.20% (*w*/*v*) alginate or k-carrageenan. Overall, the chickpea protein capsule in the presence of 0.10% (*w*/*v*) alginate offered the best protection to *B. adolescentis* in synthetic gastric juice. Poly(d,l-lactic-co-glycolic acid) (PLGA) based microcapsules, containing galacto-oligosaccharides encapsulated into an alginate–chitosan matrix along with a probiotic *B. breve* strain resulted in enhanced survival of the cells in acidic environment [166].

FOS in combination with whey protein isolate (WPI) or denatured whey protein isolate (DWPI) were also reported as carriers for probiotic strains of *L. plantarum* strains [214]. The produced microcapsules showed high encapsulation efficacy whereas particle stickiness and aggregation were reduced, along with better storage stability. Recently, Singh et al. [170] entrapped the probiotic strain *L. rhamnosus* GG in novel cellulose/chitosan-based particles supporting bacterial storage stability. The different particles also presented low toxicity to a colon CaCo-2 cell line [170]. In another study, Yao et al. [177] reported increased survival of *P. pentosaceus* after encapsulation in microgels with inorganic nanoparticles. More specifically the authors reported that the addition of MgO enhanced the viability of bacteria by filling the pores inside the microgels. Furthermore, it was observed that the presence of MgO-loaded microgels reduced the acid-induced degradation of bacteria by neutralizing the hydrogen ions in the gastric fluids [177]. Incorporation of probiotics to different food products has also been evaluated. Alginate- soy protein isolate (SPI) based hydrogel beads have been also used for incorporating *L. plantarum* cells into mango juice. Probiotics’ encapsulation showed successful resistance to thermal conditions (72 °C for 90 s) under pasteurization process [226].

#### 5.5.2. Encapsulation Technologies

An effective encapsulation process can be defined as the process by which live cells are packaged within a shell material to offer protection against unfavorable environmental conditions and allowing for their controlled release under intestinal conditions [227]. So far, several methods are available for the encapsulation of probiotics, such as spray drying, extrusion, emulsion or phase separation, freeze drying, ionotropic gelation [228] (Table 2). All these methods have several advantages and disadvantages to offer in probiotic encapsulation. Spray drying for example, can be considered as the most efficient method in terms of cost, equipment availability, particle size, processing volumes and being a continuous process [143,229,230]. However, probiotic cell viability may be significantly influenced by spray drying conditions. Several reviews describe thoroughly the different microencapsulation methods used for probiotic bacteria. Hence, only few and most recent studies will be reported. Lately, complex coacervation has been successfully applied for probiotic encapsulation [168,204,231].

A mixture of *L. acidophilus* LA-5, *B. animalis* subsp. *lactis* BB-12 and a novel potential probiotic strain *P. jensenii* 702, was spray dried (inlet temperature 195 °C and outlet temperature 85 °C) and the viability during storage was evaluated. Spray drying resulted in a significant reduction in the viability of all three probiotics. All three probiotics were able to maintain satisfactory viability levels (10^6^–10^8^ cfu/g) after spray drying, still their viability declined dramatically after storage at 30 °C. However, lactobacilli and propionibacteria remained virtually unaffected under storage at 4 °C, satisfying the recommendations regarding the level of viable cells in probiotic foods [232].

Two wall materials (native rice starch, NRS, and inulin, IN) without cross-linking agents were used as protectants of *L. rhamnosus* during spray-drying by determining the viability of the microorganism under two storage conditions [221]. The use of both colloidal prebiotics (NRS and IN) without a bonding agent was found suitable to protect *L. rhamnosus* during spray drying. The varying morphological and physicochemical properties conferred by each hydrocolloid, along with the different degree of protection provided by each of these encapsulants, suggested that the release of the microbial cells in the gastrointestinal system or in the food matrix could potentially differ depending on the wall material used in the encapsulation process. The viability and stability results indicated that both hydrocolloids protected substantially the *L. rhamnosus* cells upon spray-drying.

Fabrication of hydrogel Ca-alginate/chitosan microcapsules has been employed by Zaeim et al. [179] using electrospray techniques. In that study it was revealed that the different microcapsule matrices played an important role on survival of bacteria during storage and acid exposure. It was also presented that the outer layer of chitosan on Ca-alginate microcapsules was more efficient to protect bacteria at low pH environments [179].

Electro-spraying microencapsulation was also applied for *L. plantarum*, whereby cell viability was improved under refrigeration storage, simulation of gastric and intestinal fluids [178]. Recently, optimization of electrospraying conditions for probiotic encapsulation has been performed by Gomez-Mascaraque et al. [180]. The survival of protected *L. plantarum* evaluated during simulated in vitro gastrointestinal digestion used WPC matrix. The authors observed low viability losses and high product yields during digestion process that increased with the voltage, surfactant and prebiotic concentrations. Moreover, they presented enhanced protection of the food ingredient during storage at high relative humidity i.e., 53% and 75% [180].

## 6. Development of Synbiotics

As previously stated, the definition of prebiotic was recently restructured to “*a substrate that is selectively utilized by host microorganisms conferring a health benefit*” [134]. Hence, the definition is not limited only to fermentable carbohydrates but can also include components like polyphenols and polyunsaturated fatty acids. For instance, polyphenols might reach the colon and be further metabolized by gut microbiota [257]. Prebiotic compounds were focused to convey a health effect in the gut, reduced cardiometabolic risk and mental health (e.g., enhanced cognition). However, the new definition includes the modulation of any host microbial ecosystem [135]. Also, prebiotics will target possible health effects by modulating populations of *Roseburia*, *Eubacterium* and/or *Faecalibacterium* spp. populations apart from *Lactobacillus* and *Bifidobacterium* [135].

Fructans, FOS and inulin, as well as galacto-oligosaccharides (GOS) and lactulose, are the most commonly used compounds as prebiotics [258]. Nonetheless, an ample range of carbohydrates with different monosaccharide content and configuration of glycosidic linkages could exhibit potential prebiotic effect. Novel prebiotic compounds could derive from natural and renewable resources or synthesized enzymatically [259]. Dietary fibers and their hydrolysis products are becoming an emerging source of new ingredients with potential prebiotic activity.

Implementation of “-omic” approaches could elucidate underlying mechanisms of interaction between gut microbiota, including cross-feeding phenomena along with selectivity and specificity on fermentable carbohydrates and other components. Hence, the use of prebiotics is gaining considerable interest, as they target to sustain a healthy microbiome or restore microbial dysbiosis [260,261].

**Figure 4:**
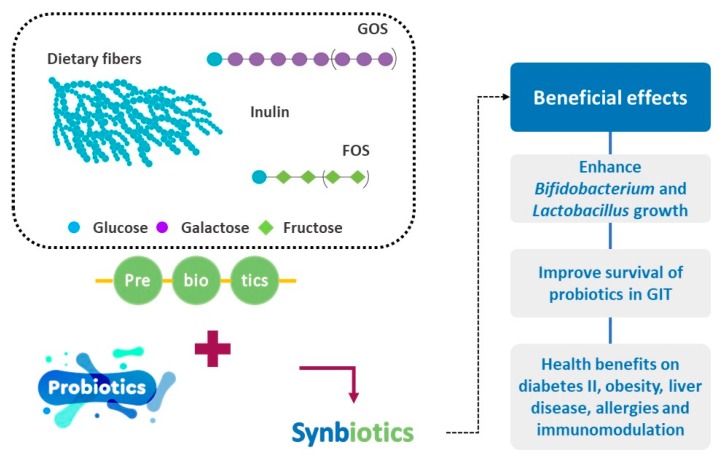
Beneficial effects of synbiotics.

A synbiotic includes the combination of a probiotic and a prebiotic and should target to enhance the survival and the implantation of the probiotic in the GIT to promote beneficial bacterial groups [262] (Figure 4). Following the definition, a synbiotic can have either complementary or synergistic action. In the first case, the prebiotic is independently selected to enhance indigenous beneficial microbiota and the probiotic is selected for a targeted biological action. On the other hand, in the latter case, the prebiotic is chosen to support specifically the growth of the selected probiotic. Hence, the prebiotic is included to be selectively fermented by the probiotic strain; regardless the beneficial impact on the population of other bacteria [262]. The development of synbiotics is emerging to be of paramount importance as it can be used as a supplement in food and nutraceutical applications. Nutraceuticals can be used as dietary supplements or functional foods, as they are food ingredients or sourced from food products that, apart from the basic original nutritional value, provide extra benefits (e.g., chronic disease prevention, improving health) [263,264,265].

Synbiotics, in a technological viewpoint, were also designed to overcome difficulties such as cell survival in the GIT. Likewise, the development of a combination in a single product could ensure an effective formulation, compared to the activity of the probiotic or prebiotic alone. Features like long-term stability during the shelf-life of food, drinks and resistance of probiotics to processing also exhibit a positive effect on the use of synbiotics.

Most synbiotic formulations include either yogurts or dairy drinks, however new products are under ongoing design. For instance, the development of synbiotic milk chocolate using encapsulated *L. casei* cells has been reported by Mandal et al. [266]. Milk chocolates presented a promising food delivery system for probiotics, whereby cell viability was enhanced with inulin. Studies conducted in vivo in mice fed with synbiotic milk chocolate led to an increase in faecal lactobacilli, decreased faecal coliforms and β-glucuronidase activity [266]. Criscio et al. [267] developed prebiotic, probiotic and synbiotic ice creams, whereby the synbiotic was formulated with inulin and *Lactobacillus* strains. Viable counts to ensure probiotic dosage were documented after frozen storage, where organoleptic characteristics were also maintained [267].

Encapsulation of food bioactives in micro- and nanoparticles via nanoscale control of food molecules could modify and enhance desired characteristics to develop functional foods. Encapsulation approaches aiming to develop synbiotics have been already applied in the literature [268]. Other novel approaches have been also presented, such as the non-dairy synbiotic beverages [269]. In that study, selected probiotic LAB were encapsulated by incorporating into rice-berry malt extract (RME). The aim was the production of a lactose free product through alginate hydrogel encapsulation including inulin. The results were quite promising since RME medium supported the growth of the selected LAB and alginate hydrogel significantly improved their survivability in the GIT. Finally, the authors suggested that the synbiotic beverage maintained high concentrations of *L. plantarun* cell under cool storage for 2 weeks.

Co-encapsulation of synbiotics usually occurs by employing alginate as a matrix. Alginate gels are stable at low pH values and can be swelled at higher pH values (as in intestinal environments) whereby release of the cells enhanced viability [270]. Atia et al., [271] studied an alginate-inulin synbiotic co-encapsulation of probiotic to target delivery in the colon as a site action. The results revealed that formulations containing inulin improved muco-adhesion properties of the probiotic beads, increasing also their protection from the acidic environment [271]. More recently synbiotic encapsulation of *L. plantarum* was also evaluated, using alginate-arabinoxylan composite microspheres, whereby encapsulation efficiency along with survival and storage stability were enhanced [268]. The same probiotic species have also been studied by Vaziri et al. [184] applying co-microencapsulation with DHA fatty acid in alginate-pectin-gelatin biocomposites.

## 7. Future Prospects

Incorporation of probiotics in foods at an industrial scale encompasses several microbiological, technological and economical challenges. Further research is required on the design of appropriate technologies, carrier matrices, and selection of bacterial strains to promote the survival of the bacterial cells under varying processing conditions (e.g., heat, osmotic and oxygen stresses) as well as during their passage through the upper gastrointestinal tract. Encapsulation of probiotic bacteria constitutes an approach that can be applied in a number of foods to achieve a wide variety of functional features. Nevertheless, several methods employed to improve probiotic viability such as microencapsulation induce an extra cost to food production process. This added manufacturing cost must be within acceptable limits to remain competitive in the globalized market of functional products.

Identifying the appropriate bacterial strains and the microencapsulation materials and process pose important challenges that need to be further addressed. It is of paramount importance that the microencapsulation methods are efficient, sustainable and environmentally friendly. Future research should focus on the optimization of probiotic cells use, considering as key factors safety and ecological production. Encapsulation materials that are not GRAS certified (e.g., chitosan) or of animal origin (e.g., gelatin) raise consumers’ concerns and remain a critical issue. On the other hand, the large size of microbial cells (1–3 μm), along with the high number of cells, can lead to large capsule sizes that may negatively influence the sensory properties of food.

Another issue that should be also considered is the safety of bacteria inclusion into food formulation, regardless that LAB strains are predominantly classified as GRAS. The origin of the strain, the delivery dose, the method and the period of supplementation, but also end users are included to assess safety. Groups of increased attention are infants, the elderly and people with compromised immune system. For instance, the expression of antibiotic resistant genes from probiotic strains combined with the indigenous gene expression of gut microbiota might result in DNA exchange between bacteria and human intestine cells as a secondary impact [272]. Ultimately, it is unequivocal that consumers should be directed towards making more conscious choices. To this end, knowledge transfer between academia, consumers, manufacturers and stakeholders is a prerequisite to avoid misinterpretation of solid scientific outcomes on the beneficial effects of probiotics.

At an industrial scale, there are several difficulties that hinder the application of microencapsulation. Different microencapsulation technologies are not yet fully exploited and require additional experimental work to be successfully implemented in real food matrices. Research efforts should be directed towards the enhancement of the properties of the microcapsules, expanding the currently available methods and overcoming the technological challenges for the production of novel functional foods. Indeed, new technical innovations are being introduced continuously. Companies using microencapsulation technologies do not hold great expectations for new, high-volume and enhanced beneficial value products. Within this context, the food industry will need further expertise to effectively introduce the most promising technologies in the evolution of the next food product generation.

On the other hand, development of synbiotics indicates a promising approach in the design of functional foods that will ultimately assist to modulate gut microbiota and convey health effects. One of the targets is to increase and maintain the cell viability after the passage through stomach and small intestine, aiming to compete with indigenous microbiota. Another major challenge will be the selection of the prebiotic candidate that will target to enhance the ability of the probiotic strain to survive in the GIT. Under this viewpoint, it is critical to investigate the selective fermentation of potential prebiotic compounds and develop novel and sustainable formulations, whereby renewable resources could be evaluated as onset materials to extract potential prebiotics.

## Figures and Tables

**Table 1:** Advances in combinations of wall materials and technologies for the microencapsulation of probiotic microorganisms.

Wall Materials	Encapsulation Technology	Microorganism	References
Whey	Agglomeration/Spray-drying	*Saccharomyces boulardii*	[167]
Whey Protein isolate/Gum Arabic	Complex coacervation	*Lactobacillus paraplantarum, Lactobacillus paracasei*	[168]
Soy protein isolate (SPI) and high methoxy pectin (HMP)	Complexation	*Lactobacillus delbrueckii*	[169]
Carboxymethyl-cellulose and chitosan	Crosslinking	*Lactobacillus rhamnosus* GG	[158]
Cellulose and chitosan	Crosslinking	*Lactobacillus rhamnosus* GG	[170]
Aguamiel, Ag, or sweet whey, SW, as inner aqueous phase	Double emulsion	*Lactobacillus plantarum*	[171]
Alginate	Dual aerosol	*Lactobacillus rhamnosus* GG and *Lactobacillus acidophilus* NCFM	[172]
Alginate and Maltodextrin	Dual aerosol/freeze drying or spray drying	*Lactobacillus rhamnosus* GG and *Lactobacillus acidophilus* NCFM	[173]
Whey protein concentrate and pullulan	Electrospinning	*Bifidobacterium animalis* subsp. *lactis* Bb12	[174]
Alginate and acidified zein	Electrospraying	*Lactobacillus acidophilus*	[175]
Alginate–human-like collagen	Electrostatic droplet generation	*Bifidobacterium longum* BIOMA 5920	[176]
Alginate-gelatin and MgO	Electrospraying	*Pediococcus pentosaceus*	[177]
Ca-alginate	Electrospraying	*Lactobacillus plantrarum*	[178]
Ca-alginate and chitosan	Electrospraying	*Lactobacillus plantrarum*	[179]
Whey protein isolate	Electrospraying	*Lactobacillus plantrarum*	[180]
Whey protein isolate/whey protein isolate and inulin/whey protein isolate and inulin and persian gum	Εlectrospraying/freeze drying/spray drying	*Lactobacillus rhamnosus* ATCC 7469	[181]
κ-carrageenan	Emulsification, freeze-drying or extrusion	*Lactobacillus plantarum*	[182]
Alginate	Emulsification/internal gelation	*Bifidobacterium bifidum* F-35	[183]
Alginate and pectin and gelatin	Emulsion	*Lactobacillus plantarum*	[184]
Chickpea protein–alginate	Emulsion	*Bifidobacterium adolescentis*	[185]
Casein, native whey and/or denatured whey proteins	Emulsion	*Lactobacillus rhamnosus* GG	[186]
Aluminum carboxymethyl cellulose–rice bran	Emulsion	*Lactobacillus reuteri*	[187]
Na- alginate (Al), alginate/1% gellan gum alginate/gum Arabic	External ionic gelation	*Lactobacillus plantarum* DKL 109	[188]
Na-alginate	Extrusion	*Lactobacillus paracasei* LAFTI^®^ L26, *Lactobacillus acidophilus* Ki, *Bifidobacterium animalis* BB-12, *Lactobacillus casei* -01	[189]
Na-alginate and chitosan	Extrusion	*Bifidobacterium pseudocatenulatum G4*	[190]
Na-alginate and fructo-oligosaccharides	Extrusion	*Lactobacillus casei* LC-01 and *Lactobacillus casei BGP* 93	[191]
Alginate-whey protein	Extrusion	*Lactobacillus delbrueckii subsp. Bulgaricus*	[192]
Alginate coated with chitosan and gelatin	Extrusion	*Lactobacillus plantarum* TN9*Lactobacillus plantarum* TN9	[193]
Legume protein isolate–alginate	Extrusion	*Bifidobacterium* *. adolescentis*	[194]
Carrageenan-locust bean gum coated milk microspheres	Extrusion	*Lactobacillus bulgaricus*	[195]
Alginate–milk	Extrusion	*Lactobacillus bulgaricus*	[196]
Alginate (ALG) and alginate-psyllium (ALG-PSL)	Extrusion	*Lactobacillus acidophilus*	[197]
Alginate/chitosan/alginate	Extrusion	*Lactobacillus salivarus*	[198]
Alginate-skim milk	Extrusion	*Lactobacillus reuteri* DPC16	[199]
Alginate–chitosan	Extrusion	*Enterococcus faecium* MC13	[200]
Whey protein isolate	Extrusion	*Lactobacillus rhamnosus* GG	[201]
Pea protein isolate–alginate	Extrusion	*Bifidobacterium adolescentis*	[202]
Na-alginate	Extrusion/emulsion	*Bifidobacterium. longum*	[164]
Na-alginate coated with starch and chitosan	Extrusion	*Lactobacillus acidophilus*	[157]
Sweet whey and shellac	Fluidized bed microencapsulation	*Lactobacillus reuteri*	[203]
Gelatin and gum Arabic	Freeze drying	*Bifidobacterium lactis*	[204]
Na-alginate, gellan gum and skim milk powder	Freeze drying	*Lactobacillus kefiranofaciens* M1	[205]
Sugarcane bagasse (SB) and sodium alginate (naa)	Immobilization/extrusion	*Lactobacillus rhamnosus* NRRL 442	[206]
Pectin coated with whey protein heat treated or without heat treatment	Ionotropic gelation and electrostatic interactions	*Lactobacillus acidophilus* La5	[207]
Chitosan and carboxymethyl cellulose	Layer by layer	*Lactobacillus acidophilus*	[208]
Chitosan and dextran sulfate	Layer-by-layer technique (lbl) using oppositely charged polyelectrolytes	*Saccharomyces boulardii*	[209]
Sodium caseinate and gellan gum	Ph- induced gelation	*Lactobacillus casei*	[210]
Solid lipid microparticles With prebiotics (inulin, polydextrose)	Spray chilling	*Lactobacillus acidophilus*	[211]
Vegetable fat with lecithin	Spray chilling	*Bifidobacterium lactis*, *Lactobacillus acidophilus*	[212]
Gum Arabic and β-cyclodextrin	Spey chilling and spray drying	*Lactobacillys acidophilus*	[213]
Fructo-oligosaccharide (FOS) and whey proteins	Spray drying	*Lactobacillus plantarum* MTCC 5422	[214]
Gum Arabic/maltodextrin/whey protein concentrate	Spray drying	*Lactobacillus acidophilus*	[159]
Slurry fermentation with whey	Spray drying	*Lactobacillus reuteri*	[215]
Skim milk and whey, maltodextrin, pectin, and arabic gum	Spray drying	*Lactobacillus plantarum*	[216]
Reconstituted skim milk (RSM) with prebiotics (inulin, oligofructose-enriched inulin, and oligofructose	Spray drying	*Bifidobacterium* BB-12	[217]
Whey	Spray drying	*Bifidobacterium* Bb-12	[218]
Whey protein isolate with sodium alginate and denatured whey protein isolate with sodium alginate	Spray drying and freeze drying	*Lactobacillus plantarum*	[219]
Sweet whey or skim milk	Spray drying	*Lactobacillus acidophilus* La-5	[220]
Native rice starch and inulin	Spray drying	*Lactobacillus rhamnosus*	[221]
Maltodextrin	Spray drying	*Lactobacillus casei*	[222]
Whey protein	Spray drying	*Lactobacilus acidophilus*, *Lactobacillus paracasei* L26 and *Bifidobacterium animalis* BB-12	[223]
Poly(vinylpyrrolidone)-poly(vinylacetate-co-crotonic acid)	Supercritical carbon dioxide	*Bifidobacterium longum* Bb46	[224]
Alginates-chitosan	Vibrating technology/extrusion	*Lactobacillus reuteri* DSM 17938	[225]

**Table 2:** Food applications of microencapsulated probiotic bacteria.

Food	Microorganism	Coating Materials	Method	References
Apple juice	*Lactobacillus rhamnosus* GG	WPI alone and in combination with a modified resistant starch (RS)	Spray drying	[233]
Carrot Juice	*Lactobacillus casei*	Chitosan-Ca-alginate	Extrusion	[234]
Carrot juice	*Lactobacillus acidophilus*	Alginate-inulin-xanthan gum	Extrusion	[235]
Cheddar cheese	*Bifidobacterium longum*	Na-alginate and palmitoylated alginate	(i) droplet extrusion method (ADE) and (ii) emulsion method	[236]
Dry fermented sausages	*Lactobacillus reuteri*	Alginate	Extrusion	[237]
Fermented milk	*Lactobacillus casei* ATCC393	Chios mastic gum	Freeze drying	[162]
Fruit juice	*Lactobacillus paracasei* L26	Alginate	Extrusion	[238]
Fruit juice	*Lactobacillus rhamnosus* GG	Whey/alginate	Droplet extrusion with coating via electrostatic deposition	[239]
Fruit juices	*Bifidobacterium longum, Bifidobacterium breve*	poly-γ-glutamic acid	Freeze drying	[240]
Fruit juices	*Lactobacillus plantarum* and *Bifidobacterium longum*	Alginate or pectin coated with chitosan, gelatin or glucomannan	Extrusion	[241]
Ice cream	*Lactobacillus casei* Lc-01 and *Bifidobacterium lactis* Bb-12	Alginate and Hi-maize resistant starch	Emulsion	[242]
Iranian yogurt drink (Doogh)	*Lactobacillus acidophilus* LA-5 and *Bifidobacterium lactis* Bb-12	Alginate	Extrusion	[243]
Kasar cheese	*Lactobacillus acidophilus* LA-5 *and Bifidobacterium bifidum* BB-12	Alginate	Emulsion or extrusion	[244]
kefir	*Bifidobacterium animals*	Sodium alginate	Extrusion	[245]
Mango juice	*Lactobacillus plantarum*	Calcium-Alginate-Soy Protein Isolate	Gelation	[226]
Mozzarella cheese	*Lactobacillus paracasei* ssp. *paracasei* LBC-1	Alginate	Extrusion	[246]
Oaxaca cheese	*Lactobacillus plantarum*	Aguamiel, Ag, or sweet whey, SW, as inner aqueous phase	Double emulsion	[171]
Pecorino cheese	*L. acidophilus*, *B. longum* and *B. lactis*	Na-alginate	Extrusion	[247]
Pecorino cheese	*Lactobacillus acidophilus* and a mix of *Bifidobacterium longum* and *Bifidobacterium lactis*	Alginate	Extrusion	[248]
Pomegranate juice	*L* *actobacillus* *plantarum*	Alginate beads coating with double layer Chitosan	Extrusion	[249]
White-brined cheese	*Bifidobacterium bifidum* BB-12 and *Lactobacillus acidophilus* LA-5	Alginate	Emulsion or extrusion	[250]
Yogurt	*Lactobacillus acidophilus* LA-5	Pectin – Whey protein	Ionic gelation and complexation	[251]
Yogurt	*Bifidobacterium bifidum* F-35	Whey/alginate	Extrusion	[252]
Yogurt	*Lactobacillus acidophilus* ATCC 4356	Alginates	Extrusion	[246]
Yogurt	*Bifidobacterium animalis* subsp. *lactis* Bb12 and *Lactobacillus rhamnosus*	Alginate	Extrusion	[253]
Yogurt	*Lactobacillus plantarum*	Sodium alginate or pectin, coated with sodium alginate or chitosan	Extrusion	[91]
Yogurt	*Lactobacillus casei*	Sodium alginate (A), amidated low-methoxyl pectin (P), and blends	Extrusion	[254]
Yogurt	*Lactobacillus acidophilus*	alginate and chitosan	Extrusion	[255]
Yogurt—Ice cream	*Lactobacillus acidophilus* La-5	Na-alginate	Extrusion	[256]

## References

[1] Savino T., Testa S., Messeni Petruzzelli A. (2018). Researcher understanding of food innovations in Nordic and Southern European countries: A systematic literature review. Trends Food Sci. Technol..

[2] Guerrero L., Claret A., Verbeke W., Sulmont-Rossé C., Hersleth M., Galanakis C.M. (2016). Chapter 5—Innovation in Traditional Food Products: Does It Make Sense?. Innovation Strategies in the Food Industry.

[3] Martin-Rios C., Demen-Meier C., Gössling S., Cornuz C. (2018). Food waste management innovations in the foodservice industry. Waste Manag..

[4] Khedkar S., Carraresi L., Bröring S. (2017). Food or pharmaceuticals? Consumers’ perception of health-related borderline products. PharmaNutrition.

[5] Brown L., Caligiuri S.P.B., Brown D., Pierce G.N. (2018). Clinical trials using functional foods provide unique challenges. J. Funct. Foods.

[6] (2019). Statista. https://www.statista.com/.

[7] FAO (2013). Guidelines for the Evaluation of Probiotics in Food: Report of a Joint FAO.

[8] Meng C., Bai C., Brown T.D., Hood L.E., Tian Q. (2018). Human Gut Microbiota and Gastrointestinal Cancer. Genom. Proteom. Bioinf..

[9] Gibson G.R., Roberfroid M.B. (1995). Dietary modulation of the human colonic microbiota: Introducing the concept of prebiotics. J. Nutr..

[10] Kristensen M., Kalkman G., Prevaes S., Tramper-Stranders G., Groot K.D.W.-D., Janssens H., Tiddens H., Van Westreenen M., Van Der Ent C., Sanders E. (2016). WS07.5 Gut microbiome in healthy children and children with cystic fibrosis during the first 18 months of life. J. Cyst. Fibros..

[11] Neffe-Skocińska K., Rzepkowska A., Szydłowska A., Kołożyn-Krajewska D. (2018). Chapter 3—Trends and Possibilities of the Use of Probiotics in Food Production. Alternative and Replacement Foods.

[12] Ambalam P., Raman M., Purama R.K., Doble M. (2016). Probiotics, prebiotics and colorectal cancer prevention. Best Pract. Res. Clin. Gastroenterol..

[13] Bautista-Gallego J., Ferrocino I., Botta C., Ercolini D., Cocolin L., Rantsiou K. (2019). Probiotic potential of a *Lactobacillus rhamnosus* cheese isolate and its effect on the fecal microbiota of healthy volunteers. Food Res. Int..

[14] Tarrah A., de Castilhos J., Rossi R.C., da Duarte V.S., Ziegler D.R., Corich V., Giacomini A. (2018). In vitro Probiotic Potential and Anti-cancer Activity of Newly Isolated Folate-Producing *Streptococcus thermophilus* Strains. Front. Microbiol..

[15] Bermúdez-Humarán L.G., Salinas E., Ortiz G.G., Ramirez-Jirano L.J., Morales J.A., Bitzer-Quintero O.K. (2019). From Probiotics to Psychobiotics: Live Beneficial Bacteria Which Act on the Brain-Gut Axis. Nutrients.

[16] Bagga D., Reichert J.L., Koschutnig K., Aigner C.S., Holzer P., Koskinen K., Moissl-Eichinger C., Schopf V. (2018). Probiotics drive gut microbiome triggering emotional brain signatures. Gut Microbes.

[17] Kim N., Yun M., Oh Y.J., Choi H.J. (2018). Mind-altering with the gut: Modulation of the gut-brain axis with probiotics. J. Microbiol..

[18] Liu L., Zhu G. (2018). Gut-Brain Axis and Mood Disorder. Front. Psychiatry.

[19] Dinan T.G., Cryan J.F. (2017). Gut-brain axis in 2016: Brain-gut-microbiota axis-mood, metabolism and behaviour. Nature reviews. Gastroenterol. Hepatol..

[20] Sarkar A., Lehto S.M., Harty S., Dinan T.G., Cryan J.F., Burnet P.W.J. (2016). Psychobiotics and the Manipulation of Bacteria-Gut-Brain Signals. Trends Neurosci..

[21] Knorr D. (1998). Technology aspects related to microorganisms in functional foods. Trends Food Sci. Technol..

[22] Karimi R., Mortazavian A.M., Da Cruz A.G. (2011). Viability of probiotic microorganisms in cheese during production and storage: A review. Dairy Sci. Technol..

[23] Saxelin M. (1997). *Lactobacillus GG*—A human probiotic strain with thorough clinical documentation. Food Rev. Intern..

[24] Wilkinson M.G. (2018). Flow cytometry as a potential method of measuring bacterial viability in probiotic products: A review. Trends Food Sci. Technol..

[25] Mohammadi R., Mortazavian A.M. (2011). Review article: Technological aspects of prebiotics in probiotic fermented milks. Food Rev. Int..

[26] Stanton C., Desmond C., Coakley M., Collins J.K., Fitzgerald G., Ross R.P. (2003). Challenges facing development of probiotic-containing functional foods. Handbook of Fermented Functional Foods.

[27] Vlasova A.N., Kandasamy S., Chattha K.S., Rajashekara G., Saif L.J. (2016). Comparison of probiotic Lactobacilli and Bifidobacteria effects, immune responses and rotavirus vaccines and infection in different host species. Vet. Immunol. Immunopathol..

[28] Holzapfel W.H. (2006). Introduction to prebiotics and probiotics. Probiotics in Food Safety and Human Health.

[29] Rivera-Espinoza Y., Gallardo-Navarro Y. (2010). Non-dairy probiotic products. Food Microbiol..

[30] Holzapfel W.H., Haberer P., Snel J., Schillinger U., Huis In’T Veld J.H.J. (1998). Overview of gut flora and probiotics. Int. J. Food Microbiol..

[31] Holzapfel W.H., Haberer P., Geisen R., Björkroth J., Schillinger U. (2001). Taxonomy and important features of probiotic microorganisms in food and nutrition. Am. J. Clin. Nutr..

[32] Bruno Biavati P.M., Whitman W.B., Kämpfer F.R.P., Trujillo M., Chun J., DeVos P., Hedlund B., Dedysh S. (2015). Bifidobacterium. Bergey’s Manual of Systematics of Archaea and Bacteria.

[33] Meena G.S., Kumar N., Majumdar G.C., Banerjee R., Meena P.K., Yadav V. (2014). Growth characteristics modeling of *Lactobacillus acidophilus* using RSM and ANN. Braz. Arch. Boil. Technol..

[34] Matejčeková Z., Liptáková D., Spodniaková S., Valík Ľ. (2016). Characterization of the growth of Lactobacillus plantarum in milk in dependence on temperature. Acta Chim. Slovaca.

[35] Da Silva A.P.R., Longhi D.A., Dalcanton F., de Aragão G.M.F. (2018). Modelling the growth of lactic acid bacteria at different temperatures. Braz. Arch. Biol. Technol..

[36] Naito Y., Uchiyama K., Takagi T. (2018). A next-generation beneficial microbe: Akkermansia muciniphila. J. Clin. Biochem. Nutr..

[37] Martin R., Miquel S., Benevides L., Bridonneau C., Robert V., Hudault S., Chain F., Berteau O., Azevedo V., Chatel J.M. (2017). Functional Characterization of Novel *Faecalibacterium prausnitzii* Strains Isolated from Healthy Volunteers: A Step Forward in the Use of *F. prausnitzii* as a Next-Generation Probiotic. Front. Microbiol..

[38] Sokol H., Pigneur B., Watterlot L., Lakhdari O., Bermudez-Humaran L.G., Gratadoux J.J., Blugeon S., Bridonneau C., Furet J.P., Corthier G. (2008). Faecalibacterium prausnitzii is an anti-inflammatory commensal bacterium identified by gut microbiota analysis of Crohn disease patients. Proc. Natl. Acad. Sci. USA.

[39] Putta S., Yarla N.S., Lakkappa D.B., Imandi S.B., Malla R.R., Chaitanya A.K., Chari B.P.V., Saka S., Vechalapu R.R., Kamal M.A., Grumezescu A.M., Holban A.M. (2018). Chapter 2—Probiotics: Supplements, Food, Pharmaceutical Industry. Therapeutic, Probiotic, and Unconventional Foods.

[40] Eor J.Y., Tan P.L., Lim S.M., Choi D.H., Yoon S.M., Yang S.Y., Kim S.H. (2019). Laxative effect of probiotic chocolate on loperamide-induced constipation in rats. Food Res. Int..

[41] Mantzourani I., Kazakos S., Terpou A., Alexopoulos A., Bezirtzoglou E., Bekatorou A., Plessas S. (2018). Potential of the Probiotic *Lactobacillus Plantarum* ATCC 14917 Strain to Produce Functional Fermented Pomegranate Juice. Foods.

[42] Tenore G.C., Caruso D., Buonomo G., D’Avino M., Ciampaglia R., Maisto M., Schisano C., Bocchino B., Novellino E. (2019). Lactofermented Annurca Apple Puree as a Functional Food Indicated for the Control of Plasma Lipid and Oxidative Amine Levels: Results from a Randomised Clinical Trial. Nutrients.

[43] Trabelsi I., Ben Slima S., Ktari N., Triki M., Abdehedi R., Abaza W., Moussa H., Abdeslam A., Ben Salah R. (2019). Incorporation of probiotic strain in raw minced beef meat: Study of textural modification, lipid and protein oxidation and color parameters during refrigerated storage. Meat Sci..

[44] Soukoulis C., Yonekura L., Gan H.-H., Behboudi-Jobbehdar S., Parmenter C., Fisk I. (2014). Probiotic edible films as a new strategy for developing functional bakery products: The case of pan bread. Food Hydrocoll..

[45] Lahtinen S.J., Gueimonde M., Ouwehand A.C., Reinikainen J.P., Salminen S.J. (2005). Probiotic bacteria may become dormant during storage. Appl. Environ. Microbiol..

[46] Galdeano C.M., Perdigon G. (2004). Role of viability of probiotic strains in their persistence in the gut and in mucosal immune stimulation. J. Appl. Microbiol..

[47] Pelletier X., Laure-Boussuge S., Donazzolo Y. (2001). Hydrogen excretion upon ingestion of dairy products in lactose-intolerant male subjects: Importance of the live flora. Eur. J. Clin. Nutr..

[48] Zou J., Dong J., Yu X. (2009). Meta-analysis: Lactobacillus containing quadruple therapy versus standard triple first-line therapy for Helicobacter pylori eradication. Helicobacter.

[49] Adams C.A. (2010). The probiotic paradox: Live and dead cells are biological response modifiers. Nutr. Res. Rev..

[50] Champagne C.P., Gomes da Cruz A., Daga M. (2018). Strategies to improve the functionality of probiotics in supplements and foods. Curr. Opin. Food Sci..

[51] Barer M.R., Tang Y.-W., Sussman M., Liu D., Poxton I., Schwartzman J. (2015). Chapter 10—Bacterial Growth, Culturability and Viability. Molecular Medical Microbiology.

[52] Grattepanche F., Lacroix C., McNeil B., Archer D., Giavasis I., Harvey L. (2013). 13—Production of viable probiotic cells. Microbial Production of Food Ingredients, Enzymes and Nutraceuticals.

[53] Dargahi N., Johnson J., Donkor O., Vasiljevic T., Apostolopoulos V. (2019). Immunomodulatory effects of probiotics: Can they be used to treat allergies and autoimmune diseases?. Maturitas.

[54] The Human Microbiome Project Consortium (2010). Structure, function and diversity of the healthy human microbiome. Nature.

[55] Mills S., Stanton C., Lane J.A., Smith G.J., Ross R.P. (2019). Precision Nutrition and the Microbiome, Part I: Current State of the Science. Nutrients.

[56] Sen P., Oresic M. (2019). Metabolic Modeling of Human Gut Microbiota on a Genome Scale: An Overview. Metabolites.

[57] Kumar H., Salminen S., Verhagen H., Rowland I., Heimbach J., Bañares S., Young T., Nomoto K., Lalonde M. (2015). Novel probiotics and prebiotics: Road to the market. Curr. Opin. Biotechnol..

[58] Rastall R.A., Gibson G.R. (2015). Recent developments in prebiotics to selectively impact beneficial microbes and promote intestinal health. Curr. Opin. Biotechnol..

[59] Lee Y.K., Salminen S. (2009). Handbook of Probiotics and Prebiotics 2009.

[60] Tripathi M.K., Giri S.K. (2014). Probiotic functional foods: Survival of probiotics during processing and storage. J. Funct. Foods.

[61] Majid I., Ahmad Nayik G., Mohammad Dar S., Nanda V. (2018). Novel food packaging technologies: Innovations and future prospective. J. Saudi Soc. Agric. Sci..

[62] Jayamanne V.S., Adams M.R. (2006). Determination of survival, identity and stress resistance of probiotic bifidobacteria in bio-yoghurts. Lett. Appl. Microbiol..

[63] Shah N.P., Ding W.K., Fallourd M.J., Leyer G. (2010). Improving the stability of probiotic bacteria in model fruit juices using vitamins and antioxidants. J. Food Sci..

[64] He J., Sakaguchi K., Suzuki T. (2012). Acquired tolerance to oxidative stress in *Bifidobacterium longum* 105-A via expression of a catalase gene. Appl. Environ. Microbiol..

[65] Teixeira P.C., Castro M.H., Malcata F.X., Kirby R.M. (1995). Survival of *Lactobacillus delbrueckii* ssp. bulgaricus following spray-drying. J. Dairy Sci..

[66] Champagne C.P., Raymond Y., Gagnon R. (2008). Viability of *Lactobacillus rhamnosus* R0011 in an apple-based fruit juice under simulated storage conditions at the consumer level. J. Food Sci..

[67] Ding W.K., Shah N.P. (2008). Survival of free and microencapsulated probiotic bacteria in orange and apple juices. Int. Food Res. J..

[68] Nualkaekul S., Salmeron I., Charalampopoulos D. (2011). Investigation of the factors influencing the survival of *Bifidobacterium longum* in model acidic solutions and fruit juices. Food Chem..

[69] Sheehan V.M., Ross P., Fitzgerald G.F. (2007). Assessing the acid tolerance and the technological robustness of probiotic cultures for fortification in fruit juices. Innov. Food Sci. Emerg. Technol..

[70] Hossain M.I., Sadekuzzaman M., Ha S.-D. (2017). Probiotics as potential alternative biocontrol agents in the agriculture and food industries: A review. Food Res. Int..

[71] de Melo Pereira G.V., de Oliveira Coelho B., Magalhães Júnior A.I., Thomaz-Soccol V., Soccol C.R. (2018). How to select a probiotic? A review and update of methods and criteria. Biotechnol. Adv..

[72] Kołożyn-Krajewska D., Dolatowski Z.J. (2012). Probiotic meat products and human nutrition. Process Biochem..

[73] Speranza B., Racioppo A., Beneduce L., Bevilacqua A., Sinigaglia M., Corbo M.R. (2017). Autochthonous lactic acid bacteria with probiotic aptitudes as starter cultures for fish-based products. Food Microbiol..

[74] Rouhi M., Sohrabvandi S., Mortazavian A.M. (2013). Probiotic Fermented Sausage: Viability of Probiotic Microorganisms and Sensory Characteristics. Crit. Rev. Food Sci..

[75] Vuyst L.D., Falony G., Leroy F. (2008). Probiotics in fermented sausages. Meat Sci..

[76] Fowler A., Toner M. (2005). Cryo-injury and biopreservation. Ann. N. Y. Acad. Sci..

[77] Gill C.O. (2006). Microbiology of frozen foods. Handbook of Frozen Food Processing and Packaging.

[78] Jay J.M., Loessner M.J., Golden D.A. (2005). Modern Food Microbiology.

[79] Santivarangkna C., Kulozik U., Foerst P. (2006). Effect of carbohydrates on the survival of *Lactobacillus helveticus* during vacuum drying. Lett. Appl. Microbiol..

[80] Santivarangkna C., Kulozik U., Foerst P. (2008). Inactivation mechanisms of lactic acid starter cultures preserved by drying processes. J. Appl. Microbiol..

[81] Schutyser M.A.I., Perdana J., Boom R.M. (2012). Single droplet drying for optimal spray drying of enzymes and probiotics. Trends Food Sci. Technol..

[82] Fonseca F., Cenard S., Passot S., Wolkers W.F., Oldenhof H. (2015). Freeze-Drying of Lactic Acid Bacteria. Cryopreservation and Freeze-Drying Protocols.

[83] Fu N., Huang S., Xiao J., Chen X.D., Toldrá F. (2018). Chapter Six—Producing powders containing active dry probiotics with the aid of spray drying. Advances in Food and Nutrition Research.

[84] Bosnea L.A., Kourkoutas Y., Albantaki N., Tzia C., Koutinas A.A., Kanellaki M. (2009). Functionality of freeze-dried *L. casei* cells immobilized on wheat grains. LWT Food Sci. Technol..

[85] Terpou A., Gialleli A.-I., Bekatorou A., Dimitrellou D., Ganatsios V., Barouni E., Koutinas A.A., Kanellaki M. (2017). Sour milk production by wheat bran supported probiotic biocatalyst as starter culture. Food Bioprod. Process.

[86] Ouwehand A.C., Kirjavainen P.V., Shortt C., Salminen S. (1999). Probiotics: Mechanisms and established effects. Int. Dairy J..

[87] O’Brien J., Crittenden R., Ouwehand A.C., Salminen S. (1999). Safety evaluation of probiotics. Trends Food Sci. Technol..

[88] Sanders M.E., Klaenhammer T.R., Ouwehand A.C., Pot B., Johansen E., Heimbach J.T., Marco M.L., Tennilä J., Ross R.P., Franz C. (2014). Effects of genetic, processing, or product formulation changes on efficacy and safety of probiotics. Ann. N. Y. Acad. Sci..

[89] Hansen L.T., Allan-Wojtas P.M., Jin Y.L., Paulson A.T. (2002). Survival of Ca-alginate microencapsulated *Bifidobacterium* spp. in milk and simulated gastrointestinal conditions. Food Microbiol..

[90] Sabikhi L., Babu R., Thompkinson D.K., Kapila S. (2010). Resistance of microencapsulated *Lactobacillus acidophilus* LA1 to processing treatments and simulated gut conditions. Food Bioprocess Technol..

[91] Brinques G.B., Ayub M.A.Z. (2011). Effect of microencapsulation on survival of *Lactobacillus plantarum* in simulated gastrointestinal conditions, refrigeration, and yogurt. J. Food Eng..

[92] Vijayakumar M., Ilavenil S., Kim D.H., Arasu M.V., Priya K., Choi K.C. (2015). In-vitro assessment of the probiotic potential of *Lactobacillus plantarum* KCC-24 isolated from Italian rye-grass (*Lolium multiflorum*) forage. Anaerobe.

[93] Turková K., Mavrič A., Narat M., Rittich B., Španová A., Rogelj I., Matijašić B. (2013). Evaluation of Lactobacillus strains for selected probiotic properties. Folia Microbiol..

[94] Ranadheera C.S., Evans C.A., Adams M.C., Baines S.K. (2012). In vitro analysis of gastrointestinal tolerance and intestinal cell adhesion of probiotics in goat’s milk ice cream and yogurt. Food Res. Int..

[95] Terpou A., Bekatorou A., Kanellaki M., Koutinas A.A., Nigam P. (2017). Enhanced probiotic viability and aromatic profile of yogurts produced using wheat bran (*Triticum aestivum*) as cell immobilization carrier. Process Biochem..

[96] Terpou A., Papadaki A., Bosnea L., Kanellaki M., Kopsahelis N. (2019). Novel frozen yogurt production fortified with sea buckthorn berries and probiotics. LWT Food Sci. Technol..

[97] Clark P.A., Cotton L.N., Martin J.H. (1993). Selection of bifidobacteria for use as dietary adjuncts in cultured dairy foods: II—Tolerance to simulated pH of human stomachs. Cult. Dairy Prod. J..

[98] Liong M.T., Shah N.P. (2005). Acid and bile tolerance and cholesterol removal ability of lactobacilli strains. J. Dairy Sci..

[99] Lo Curto A., Pitino I., Mandalari G., Dainty J.R., Faulks R.M., John Wickham M.S. (2011). Survival of probiotic lactobacilli in the upper gastrointestinal tract using an in vitro gastric model of digestion. Food Microbiol..

[100] Muruzović M.Ž., Mladenović K.G., Čomić L.R. (2018). In vitro evaluation of resistance to environmental stress by planktonic and biofilm form of lactic acid bacteria isolated from traditionally made cheese from Serbia. Food Biosci..

[101] Beales N. (2004). Adaptation of microorganisms to cold temperatures, weak acid preservatives, low pH, and osmotic stress: A review. Compr. Rev. Food Sci. Food Saf..

[102] Upadrasta A., O’Sullivan L., O’Sullivan O., Sexton N., Lawlor P.G., Hill C., Fitzgerald G.F., Stanton C., Ross R.P. (2013). The Effect of Dietary Supplementation with Spent Cider Yeast on the Swine Distal Gut Microbiome. PLoS ONE.

[103] Saarela M., Rantala M., Hallamaa K., Nohynek L., Virkajärvi I., Mättö J. (2004). Stationary-phase acid and heat treatments for improvement of the viability of probiotic lactobacilli and bifidobacteria. J. Appl. Microbiol..

[104] Pénicaud C., Monclus V., Perret B., Passot S., Fonseca F. (2018). Life cycle assessment of the production of stabilized lactic acid bacteria for the environmentally-friendly preservation of living cells. J. Clean. Prod..

[105] Sauer M., Russmayer H., Grabherr R., Peterbauer C.K., Marx H. (2017). The Efficient Clade: Lactic Acid Bacteria for Industrial Chemical Production. Trends Biotechnol..

[106] Ruiz L., Ruas-Madiedo P., Gueimonde M., De Los Reyes-Gavilán C.G., Margolles A., Sánchez B. (2011). How do bifidobacteria counteract environmental challenges? Mechanisms involved and physiological consequences. Genes Nutr..

[107] Mohammadi R., Sohrabvandi S., Mohammad Mortazavian A. (2012). The starter culture characteristics of probiotic microorganisms in fermented milks. Eng. Life Sci..

[108] Bron P.A., Marcelli B., Mulder J., van der Els S., Morawska L.P., Kuipers O.P., Kok J., Kleerebezem M. (2019). Renaissance of traditional DNA transfer strategies for improvement of industrial lactic acid bacteria. Curr. Opin. Biotechnol..

[109] Panoff J.M., Thammavongs B., Guéguen M. (2000). Cryoprotectants lead to phenotypic adaptation to freeze-thaw stress in *Lactobacillus delbrueckii* ssp. *bulgaricus* CIP 101027T. Cryobiology.

[110] Gouesbet G., Jan G., Boyaval P. (2001). *Lactobacillus delbrueckii* ssp. *bulgaricus* thermotolerance. Lait.

[111] Conrad P.B., Miller D.P., Cielenski P.R., De Pablo J.J. (2000). Stabilization and preservation of *Lactobacillus acidophilus* in saccharide matrices. Cryobiology.

[112] Parkar S.G., Redgate E.L., McGhie T.K., Hurst R.D. (2014). In vitro studies of modulation of pathogenic and probiotic bacterial proliferation and adhesion to intestinal cells by blackcurrant juices. J. Funct. Foods.

[113] Van de Guchte M., Serror P., Chervaux C., Smokvina T., Ehrlich S.D., Maguin E. (2002). Stress responses in lactic acid bacteria. Antonie van Leeuwenhoek Int. J. Gen. Mol. Microbiol..

[114] De Angelis M., Gobbetti M. (2004). Environmental stress responses in Lactobacillus: A review. Proteomics.

[115] Serrazanetti D.I., Guerzoni M.E., Corsetti A., Vogel R. (2009). Metabolic impact and potential exploitation of the stress reactions in lactobacilli. Food Microbiol..

[116] Alonso García E., Pérez Montoro B., Benomar N., Castillo-Gutiérrez S., Estudillo-Martínez M.D., Knapp C.W., Abriouel H. (2019). New insights into the molecular effects and probiotic properties of *Lactobacillus pentosus* pre-adapted to edible oils. LWT Food Sci. Technol..

[117] Capozzi V., Arena M.P., Russo P., Spano G., Fiocco D., Watson R.R., Preedy V.R. (2016). Chapter 16—Stressors and Food Environment: Toward Strategies to Improve Robustness and Stress Tolerance in Probiotics. Probiotics, Prebiotics, and Synbiotics.

[118] Settachaimongkon S., van Valenberg H.J.F., Winata V., Wang X., Nout M.J.R., van Hooijdonk T.C.M., Zwietering M.H., Smid E.J. (2015). Effect of sublethal preculturing on the survival of probiotics and metabolite formation in set-yoghurt. Food Microbiol..

[119] Sunny-Roberts E.O., Ananta E., Knorr D. (2007). Flow cytometry assessment of *Lactobacillus rhamnosus* GG (ATCC 53103) response to non-electrolytes stress. Nutr. Food Sci..

[120] Gandhi A., Shah N.P. (2015). Effect of salt on cell viability and membrane integrity of *Lactobacillus acidophilus*, *Lactobacillus casei* and *Bifidobacterium longum* as observed by flow cytometry. Food Microbiol..

[121] Shah N.P. (2000). Probiotic bacteria: Selective enumeration and survival in dairy foods. J. Dairy Sci..

[122] Ahn J.B., Hwang H.J., Park J.H. (2001). Physiological responses of oxygen-tolerant anaerobic *Bifidobacterium longum* under oxygen. J. Microbiol. Biotechnol..

[123] Talwalkar A., Kailasapathy K. (2004). The role of oxygen in the viability of probiotic bacteria with reference to *L. acidophilus* and *Bifidobacterium* spp.. Curr. Issues Intest. Microbiol..

[124] Chen M.-J., Tang H.-Y., Chiang M.-L. (2017). Effects of heat, cold, acid and bile salt adaptations on the stress tolerance and protein expression of kefir-isolated probiotic *Lactobacillus kefiranofaciens* M1. Food Microbiol..

[125] Pérez Montoro B., Benomar N., Caballero Gómez N., Ennahar S., Horvatovich P., Knapp C.W., Gálvez A., Abriouel H. (2018). Proteomic analysis of *Lactobacillus pentosus* for the identification of potential markers involved in acid resistance and their influence on other probiotic features. Food Microbiol..

[126] Desmond C., Stanton C., Fitzgerald G.F., Collins K., Paul Ross R. (2001). Environmental adaptation of probiotic lactobacilli towards improvement of performance during spray drying. Int. Dairy J..

[127] Desmond C., Ross R.P., O’Callaghan E., Fitzgerald G., Stanton C. (2002). Improved survival of *Lactobacillus paracasei* NFBC 338 in spray-dried powders containing gum acacia. J. Appl. Microbiol..

[128] Ananta E., Knorr D. (2004). Evidence on the role of protein biosynthesis in the induction of heat tolerance of *Lactobacillus rhamnosus* GG by pressure pre-treatment. Int. J. Food Microbiol..

[129] Teixeira P., Castro H., Kirby R. (1994). Inducible thermotolerance in *Lactobacillus bulgaricus*. Lett. Appl. Microbiol..

[130] Korbekandi H., Mortazavian A.M., Iravani S. (2010). Technology and stability of probiotic in fermented milks. Probiotic and Prebiotic Foods: Technology, Stability and Benefits to the Human Health 2010.

[131] da Cruz A.G., de Faria J.A.F., Van Dender A.G.F. (2007). Packaging system and probiotic dairy foods. Food Res. Int..

[132] Miller C.W., Nguyen M.H., Rooney M., Kailasapathy K. (2002). The influence of packaging materials on the dissolved oxygen content of probiotic yoghurt. Packag. Technol. Sci..

[133] Miller C.W., Nguyen M.H., Rooney M., Kailasapathy K. (2003). The control of dissolved oxygen content in probiotic yoghurts by alternative packaging materials. Packag. Technol. Sci..

[134] Cruz A.G., Castro W.F., Faria J.A.F., Bolini H.M.A., Celeghini R.M.S., Raices R.S.L., Oliveira C.A.F., Freitas M.Q., Conte Júnior C.A., Mársico E.T. (2013). Stability of probiotic yogurt added with glucose oxidase in plastic materials with different permeability oxygen rates during the refrigerated storage. Food Res. Int..

[135] Gibson G.R., Hutkins R., Sanders M.E., Prescott S.L., Reimer R.A., Salminen S.J., Scott K., Stanton C., Swanson K.S., Cani P.D. (2017). Expert consensus document: The International Scientific Association for Probiotics and Prebiotics (ISAPP) consensus statement on the definition and scope of prebiotics. Nature reviews. Gastroenterol. Hepatol..

[136] Tian Q., Wang T.-T., Tang X., Han M.-Z., Leng X.-J., Mao X.-Y. (2015). Developing a potential prebiotic of yogurt: Growth of Bifidobacterium and yogurt cultures with addition of glycomacropeptide hydrolysate. Int. J. Food Sci. Technol..

[137] Shin H.S., Lee J.H., Pestka J.J., Ustunol Z. (2000). Growth and viability of commercial *Bifidobacterium* spp in skim milk containing oligosaccharides and inulin. J. Food Sci..

[138] Capela P., Hay T.K.C., Shah N.P. (2006). Effect of cryoprotectants, prebiotics and microencapsulation on survival of probiotic organisms in yoghurt and freeze-dried yoghurt. Food Res. Int..

[139] McComas K.A., Gilliland S.E. (2003). Growth of Probiotic and Traditional Yogurt Cultures in Milk Supplemented with Whey Protein Hydrolysate. J. Food Sci..

[140] Carvalho A.S., Silva J., Ho P., Teixeira P., Malcata F.X., Gibbs P. (2004). Effects of Various Sugars Added to Growth and Drying Media upon Thermotolerance and Survival throughout Storage of Freeze-Dried *Lactobacillus delbrueckii* ssp. *bulgaricus*. Biotechnol. Prog..

[141] Dave R.I., Shah N.P. (1998). Ingredient Supplementation Effects on Viability of Probiotic Bacteria in Yogurt. J. Dairy Sci..

[142] Akalin A.S., Fenderya S., Akbulut N. (2004). Viability and activity of bifidobacteria in yoghurt containing fructooligosaccharide during refrigerated storage. Int. J. Food Sci. Technol..

[143] Ananta E., Volkert M., Knorr D. (2005). Cellular injuries and storage stability of spray-dried *Lactobacillus rhamnosus* GG. Int. Dairy J..

[144] Cordeiro B.F., Oliveira E.R., da Silva S.H., Savassi B.M., Acurcio L.B., Lemos L., Alves J.L., Carvalho Assis H., Vieira A.T., Faria A.M.C. (2018). Whey Protein Isolate-Supplemented Beverage, Fermented by *Lactobacillus casei* BL23 and Propionibacterium freudenreichii 138, in the Prevention of Mucositis in Mice. Front. Microbiol..

[145] Su J., Wang X., Li W., Chen L., Zeng X., Huang Q., Hu B. (2018). Enhancing the Viability of Lactobacillus plantarum as Probiotics through Encapsulation with High Internal Phase Emulsions Stabilized with Whey Protein Isolate Microgels. J. Agric. Food Chem..

[146] Guo M., Yadav M.P., Jin T.Z. (2017). Antimicrobial edible coatings and films from micro-emulsions and their food applications. Int. J. Food Microbiol..

[147] Bambace M.F., Alvarez M.V., del Moreira M.R. (2019). Novel functional blueberries: Fructo-oligosaccharides and probiotic lactobacilli incorporated into alginate edible coatings. Food Res. Int..

[148] Ebrahimi B., Mohammadi R., Rouhi M., Mortazavian A.M., Shojaee-Aliabadi S., Koushki M.R. (2018). Survival of probiotic bacteria in carboxymethyl cellulose-based edible film and assessment of quality parameters. LWT Food Sci. Technol..

[149] Soukoulis C., Behboudi-Jobbehdar S., Macnaughtan W., Parmenter C., Fisk I.D. (2017). Stability of *Lactobacillus rhamnosus* GG incorporated in edible films: Impact of anionic biopolymers and whey protein concentrate. Food Hydrocoll..

[150] Pavli F., Kovaiou I., Apostolakopoulou G., Kapetanakou A., Skandamis P., Nychas G.E., Tassou C., Chorianopoulos N. (2017). Alginate-Based Edible Films Delivering Probiotic Bacteria to Sliced Ham Pretreated with High Pressure Processing. Int. J. Mol. Sci..

[151] Ningtyas D.W., Bhandari B., Bansal N., Prakash S. (2019). The viability of probiotic *Lactobacillus rhamnosus* (non-encapsulated and encapsulated) in functional reduced-fat cream cheese and its textural properties during storage. Food Control.

[152] Kailasapathy K. (2003). Protecting probiotic by microencapsulation. Microbial. Aust..

[153] Lee K.Y., Heo T.R. (2000). Survival of *Bifidobacterium longum* immobilized in calcium alginate beads in simulated gastric juices and bile salt solution. Appl. Environ. Microbiol..

[154] Muthukumarasamy P., Allan-Wojtas P., Holley R.A. (2006). Stability of *Lactobacillus reuteri* in different types of microcapsules. J. Food Sci..

[155] Cabuk B., Harsa S.T. (2015). Improved viability of *Lactobacillus acidophilus* NRRL-B 4495 during freeze-drying in whey protein-pullulan microcapsules. J. Microencapsul..

[156] Călinoiu L.-F., Ştefănescu B.E., Pop I.D., Muntean L., Vodnar D.C. (2019). Chitosan Coating Applications in Probiotic Microencapsulation. Coatings.

[157] de Araújo Etchepare M., Raddatz G.C., de Moraes Flores É.M., Zepka L.Q., Jacob-Lopes E., Barin J.S., Ferreira Grosso C.R., de Menezes C.R. (2016). Effect of resistant starch and chitosan on survival of *Lactobacillus acidophilus* microencapsulated with sodium alginate. LWT Food Sci. Technol..

[158] Singh P., Medronho B., Alves L., da Silva G.J., Miguel M.G., Lindman B. (2017). Development of carboxymethyl cellulose-chitosan hybrid micro-and macroparticles for encapsulation of probiotic bacteria. Carbohydr. Polym..

[159] Colín-Cruz M.A., Pimentel-González D.J., Carrillo-Navas H., Alvarez-Ramírez J., Guadarrama-Lezama A.Y. (2019). Co-encapsulation of bioactive compounds from blackberry juice and probiotic bacteria in biopolymeric matrices. LWT Food Sci. Technol..

[160] Vega-Sagardía M., Rocha J., Sáez K., Smith C.T., Gutierrez-Zamorano C., García-Cancino A. (2018). Encapsulation, with and without oil, of biofilm forming Lactobacillus fermentum UCO-979C strain in alginate-xanthan gum and its anti-Helicobacter pylori effect. J. Funct. Foods.

[161] Schoina V., Terpou A., Bosnea L., Kanellaki M., Nigam P.S. (2018). Entrapment *of Lactobacillus casei* ATCC393 in the viscus matrix of Pistacia terebinthus resin for functional myzithra cheese manufacture. LWT Food Sci. Technol..

[162] Terpou A., Nigam P.S., Bosnea L., Kanellaki M. (2018). Evaluation of Chios mastic gum as antimicrobial agent and matrix forming material targeting probiotic cell encapsulation for functional fermented milk production. LWT Food Sci. Technol..

[163] Heidebach T., Först P., Kulozik U. (2010). Influence of casein-based microencapsulation on freeze-drying and storage of probiotic cells. J. Food Eng..

[164] Phoem A.N., Chanthachum S., Voravuthikunchai S.P. (2015). Preparation of eleutherine americana-alginate complex microcapsules and application in *Bifidobacterium longum*. Nutrients.

[165] Thantsha M.S., Labuschagne P.W., Mamvura C.I. (2014). Supercritical CO2 interpolymer complex encapsulation improves heat stability of probiotic bifidobacteria. World J. Microbiol. Biotechnol..

[166] Cook M.T., Tzortzis G., Charalampopoulos D., Khutoryanskiy V.V. (2014). Microencapsulation of a synbiotic into PLGA/alginate multiparticulate gels. Int. J. Pharm..

[167] Duongthingoc D., George P., Katopo L., Gorczyca E., Kasapis S. (2013). Effect of whey protein agglomeration on spray dried microcapsules containing *Saccharomyces boulardii*. Food Chem..

[168] Bosnea L.A., Moschakis T., Biliaderis C.G. (2014). Complex Coacervation as a Novel Microencapsulation Technique to Improve Viability of Probiotics Under Different Stresses. Food Bioprocess Technol..

[169] Sun Q., Wang F., Han D., Zhao Y., Liu Z., Lei H., Song Y., Huang X., Li X., Ma A. (2014). Preparation and optimization of soy protein isolate–high methoxy pectin microcapsules loaded with *Lactobacillus delbrueckii*. Int. J. Food Sci. Technol..

[170] Singh P., Medronho B., dos Santos T., Nunes-Correia I., Granja P., Miguel M.G., Lindman B. (2018). On the viability, cytotoxicity and stability of probiotic bacteria entrapped in cellulose-based particles. Food Hydrocoll..

[171] Rodríguez-Huezo M.E., Estrada-Fernández A.G., García-Almendárez B.E., Ludeña-Urquizo F., Campos-Montiel R.G., Pimentel-González D.J. (2014). Viability of *Lactobacillus plantarum* entrapped in double emulsion during Oaxaca cheese manufacture, melting and simulated intestinal conditions. LWT Food Sci. Technol..

[172] Sohail A., Turner M.S., Coombes A., Bostrom T., Bhandari B. (2011). Survivability of probiotics encapsulated in alginate gel microbeads using a novel impinging aerosols method. Int. J. Food Microbiol..

[173] Sohail A., Turner M., Coombes A., Bhandari B. (2013). The Viability of *Lactobacillus rhamnosus* GG and *Lactobacillus acidophilus* NCFM Following Double Encapsulation in Alginate and Maltodextrin. Food Bioprocess Technol..

[174] López-Rubio A., Sanchez E., Wilkanowicz S., Sanz Y., Lagaron J.M. (2012). Electrospinning as a useful technique for the encapsulation of living bifidobacteria in food hydrocolloids. Food Hydrocoll..

[175] Laelorspoen N., Wongsasulak S., Yoovidhya T., Devahastin S. (2014). Microencapsulation of *Lactobacillus acidophilus* in zein–alginate core–shell microcapsules via electrospraying. J. Funct. Foods.

[176] Su R., Zhu X.-L., Fan D.-D., Mi Y., Yang C.-Y., Jia X. (2011). Encapsulation of probiotic *Bifidobacterium longum* BIOMA 5920 with alginate–human-like collagen and evaluation of survival in simulated gastrointestinal conditions. Int. J. Biol. Macromol..

[177] Yao M., Li B., Ye H., Huang W., Luo Q., Xiao H., McClements D.J., Li L. (2018). Enhanced viability of probiotics (*Pediococcus pentosaceus* Li05) by encapsulation in microgels doped with inorganic nanoparticles. Food Hydrocoll..

[178] Coghetto C.C., Flores S.H., Brinques G.B., Záchia Ayub M.A. (2016). Viability and alternative uses of a dried powder, microencapsulated *Lactobacillus plantarum* without the use of cold chain or dairy products. LWT Food Sci. Technol..

[179] Zaeim D., Sarabi-Jamab M., Ghorani B., Kadkhodaee R., Tromp R.H. (2017). Electrospray assisted fabrication of hydrogel microcapsules by single-and double-stage procedures for encapsulation of probiotics. Food Bioprod. Process.

[180] Gomez-Mascaraque L.G., Morfin R.C., Pérez-Masiá R., Sanchez G., Lopez-Rubio A. (2016). Optimization of electrospraying conditions for the microencapsulation of probiotics and evaluation of their resistance during storage and in-vitro digestion. LWT Food Sci. Technol..

[181] Moayyedi M., Eskandari M.H., Rad A.H.E., Ziaee E., Khodaparast M.H.H., Golmakani M.-T. (2018). Effect of drying methods (electrospraying, freeze drying and spray drying) on survival and viability of microencapsulated *Lactobacillus rhamnosus* ATCC 7469. J. Funct. Foods.

[182] Tee W.F., Nazaruddin R., Tan Y.N., Ayob M.K. (2014). Effects of encapsulation on the viability of potential probiotic *Lactobacillus plantarum* exposed to high acidity condition and presence of bile salts. Food Sci. Technol. Int..

[183] Zou Q., Zhao J., Liu X., Tian F., Zhang H.P., Zhang H., Chen W. (2011). Microencapsulation of *Bifidobacterium bifidum* F-35 in reinforced alginate microspheres prepared by emulsification/internal gelation. Int. J. Food Sci. Technol..

[184] Vaziri A.S., Alemzadeh I., Vossoughi M., Khorasani A.C. (2018). Co-microencapsulation of *Lactobacillus plantarum* and DHA fatty acid in alginate-pectin-gelatin biocomposites. Carbohydr. Polym..

[185] Wang J., Korber D.R., Low N.H., Nickerson M.T. (2014). Entrapment, survival and release of *Bifidobacterium adolescentis* within chickpea protein-based microcapsules. Food Res. Int..

[186] Burgain J., Gaiani C., Cailliez-Grimal C., Jeandel C., Scher J. (2013). Encapsulation of *Lactobacillus rhamnosus* GG in microparticles: Influence of casein to whey protein ratio on bacterial survival during digestion. Innov. Food Sci. Emerg. Technol..

[187] Chitprasert P., Sudsai P., Rodklongtan A. (2012). Aluminum carboxymethyl cellulose–rice bran microcapsules: Enhancing survival of *Lactobacillus reuteri* KUB-AC5. Carbohydr. Polym..

[188] Chun H., Kim C.H., Cho Y.H. (2014). Microencapsulation of *Lactobacillus plantarum* DKL 109 using External Ionic Gelation Method. Korean J. Food Sci. Anim. Resour..

[189] Sousa S., Gomes A.M., Pintado M.M., Silva J.P., Costa P., Amaral M.H., Duarte A.C., Rodrigues D., Rocha-Santos T.A.P., Freitas A.C. (2015). Characterization of freezing effect upon stability of, probiotic loaded, calcium-alginate microparticles. Food Bioprod. Process.

[190] Kamalian N., Mirhosseini H., Mustafa S., Manap M.Y.A. (2014). Effect of alginate and chitosan on viability and release behavior of *Bifidobacterium pseudocatenulatum* G4 in simulated gastrointestinal fluid. Carbohydr. Polym..

[191] Rodrigues F.J., Omura M.H., Cedran M.F., Dekker R.F.H., Barbosa-Dekker A.M., Garcia S. (2017). Effect of natural polymers on the survival of *Lactobacillus casei* encapsulated in alginate microspheres. J. Microencapsul..

[192] Chen M.Y., Zheng W., Dong Q.Y., Li Z.H., Shi L.E., Tang Z.X. (2014). Activity of Encapsulated *Lactobacillus bulgaricus* in Alginate-whey Protein Microspheres. Braz. Arch. Biol. Technol..

[193] Trabelsi I., Ayadi D., Bejar W., Bejar S., Chouayekh H., Ben Salah R. (2014). Effects of *Lactobacillus plantarum* immobilization in alginate coated with chitosan and gelatin on antibacterial activity. Int. J. Biol. Macromol..

[194] Khan N.H., Korber D.R., Low N.H., Nickerson M.T. (2013). Development of extrusion-based legume protein isolate–alginate capsules for the protection and delivery of the acid sensitive probiotic, *Bifidobacterium adolescentis*. Food Res. Int..

[195] Shi L.-E., Li Z.-H., Zhang Z.-L., Zhang T.-T., Yu W.-M., Zhou M.-L., Tang Z.-X. (2013). Encapsulation of *Lactobacillus bulgaricus* in carrageenan-locust bean gum coated milk microspheres with double layer structure. LWT Food Sci. Technol..

[196] Shi L.-E., Li Z.-H., Li D.-T., Xu M., Chen H.-Y., Zhang Z.-L., Tang Z.-X. (2013). Encapsulation of probiotic *Lactobacillus bulgaricus* in alginate–milk microspheres and evaluation of the survival in simulated gastrointestinal conditions. J. Food Eng..

[197] Lotfipour F., Mirzaeei S., Maghsoodi M. (2012). Preparation and Characterization of Alginate and Psyllium Beads Containing *Lactobacillus acidophilus*. Sci. World J..

[198] Bajracharya P., Islam M.A., Jiang T., Kang S.-K., Choi Y.-J., Cho C.-S. (2012). Effect of microencapsulation of *Lactobacillus salivarus* 29 into alginate/chitosan/alginate microcapsules on viability and cytokine induction. J. Microencapsul..

[199] Zhao Q., Mutukumira A., Lee S., Maddox I., Shu Q. (2012). Functional properties of free and encapsulated *Lactobacillus reuteri* DPC16 during and after passage through a simulated gastrointestinal tract. World J. Microbiol. Biotechnol..

[200] Kanmani P., Satish Kumar R., Yuvaraj N., Paari K.A., Pattukumar V., Arul V. (2011). Effect of cryopreservation and microencapsulation of lactic acid bacterium *Enterococcus faecium* MC13 for long-term storage. Biochem. Eng. J..

[201] Doherty S.B., Gee V.L., Ross R.P., Stanton C., Fitzgerald G.F., Brodkorb A. (2011). Development and characterisation of whey protein micro-beads as potential matrices for probiotic protection. Food Hydrocoll..

[202] Klemmer K.J., Korber D.R., Low N.H., Nickerson M.T. (2011). Pea protein-based capsules for probiotic and prebiotic delivery. Int. J. Food Sci. Technol..

[203] Schell D., Beermann C. (2014). Fluidized bed microencapsulation of *Lactobacillus reuteri* with sweet whey and shellac for improved acid resistance and in-vitro gastro-intestinal survival. Food Res. Int..

[204] Marques da Silva T., Jacob Lopes E., Codevilla C.F., Cichoski A.J., Flores É.M.d.M., Motta M.H., da Silva C.B., Grosso C.R.F., de Menezes C.R. (2018). Development and characterization of microcapsules containing *Bifidobacterium* Bb-12 produced by complex coacervation followed by freeze drying. LWT Food Sci. Technol..

[205] Wang S.Y., Ho Y.F., Chen Y.P., Chen M.J. (2015). Effects of a novel encapsulating technique on the temperature tolerance and anti-colitis activity of the probiotic bacterium Lactobacillus kefiranofaciens M1. Food Microbiol..

[206] Shaharuddin S., Muhamad I.I. (2015). Microencapsulation of alginate-immobilized bagasse with *Lactobacillus rhamnosus* NRRL 442: Enhancement of survivability and thermotolerance. Carbohydr. Polym..

[207] Gebara C., Chaves K.S., Ribeiro M.C.E., Souza F.N., Grosso C.R.F., Gigante M.L. (2013). Viability of *Lactobacillus acidophilus* La5 in pectin–whey protein microparticles during exposure to simulated gastrointestinal conditions. Food Res. Int..

[208] Priya A.J., Vijayalakshmi S.P., Raichur A.M. (2011). Enhanced Survival of Probiotic *Lactobacillus acidophilus* by Encapsulation with Nanostructured Polyelectrolyte Layers through Layer-by-Layer Approach. J. Agric. Food Chem..

[209] Thomas M.B., Vaidyanathan M., Radhakrishnan K., Raichur A.M. (2014). Enhanced viability of probiotic *Saccharomyces boulardii* encapsulated by layer-by-layer approach in pH responsive chitosan-dextran sulfate polyelectrolytes. J. Food Eng..

[210] Nag A., Han K.-S., Singh H. (2011). Microencapsulation of probiotic bacteria using pH-induced gelation of sodium caseinate and gellan gum. Int. Dairy J..

[211] Okuro P.K., Thomazini M., Balieiro J.C.C., Liberal R.D., Fávaro-Trindade C.S. (2013). Co-encapsulation of *Lactobacillus acidophilus* with inulin or polydextrose in solid lipid microparticles provides protection and improves stability. Food Res. Int..

[212] de Pedroso D.L., Thomazini M., Heinemann R.J.B., Favaro-Trindade C.S. (2012). Protection of *Bifidobacterium lactis* and *Lactobacillus acidophilus* by microencapsulation using spray-chilling. Int. Dairy J..

[213] Arslan-Tontul S., Erbas M. (2017). Single and double layered microencapsulation of probiotics by spray drying and spray chilling. LWT Food Sci. Technol..

[214] Rajam R., Anandharamakrishnan C. (2015). Microencapsulation of *Lactobacillus plantarum* (MTCC 5422) with fructooligosaccharide as wall material by spray drying. LWT Food Sci. Technol..

[215] Jantzen M., Göpel A., Beermann C. (2013). Direct spray drying and microencapsulation of probiotic *Lactobacillus reuteri* from slurry fermentation with whey. J. Appl. Microbiol..

[216] Bustos P., Bórquez R. (2013). Influence of Osmotic Stress and Encapsulating Materials on the Stability of Autochthonous *Lactobacillus plantarum* after Spray Drying. Dry Technol..

[217] Fritzen-Freire C.B., Prudêncio E.S., Pinto S.S., Muñoz I.B., Amboni R.D.M.C. (2013). Effect of microencapsulation on survival of *Bifidobacterium* BB-12 exposed to simulated gastrointestinal conditions and heat treatments. LWT Food Sci. Technol..

[218] De Castro-Cislaghi F.P., Silva C.D.R.E., Fritzen-Freire C.B., Lorenz J.G., Sant’Anna E.S. (2012). *Bifidobacterium* Bb-12 microencapsulated by spray drying with whey: Survival under simulated gastrointestinal conditions, tolerance to NaCl, and viability during storage. J. Food Eng..

[219] Rajam R., Karthik P., Parthasarathi S., Joseph G.S., Anandharamakrishnan C. (2012). Effect of whey protein–alginate wall systems on survival of microencapsulated *Lactobacillus plantarum* in simulated gastrointestinal conditions. J. Funct. Foods.

[220] Maciel G.M., Chaves K.S., Grosso C.R.F., Gigante M.L. (2014). Microencapsulation of *Lactobacillus acidophilus* La-5 by spray-drying using sweet whey and skim milk as encapsulating materials. J. Dairy Sci..

[221] Avila-Reyes S.V., Garcia-Suarez F.J., Jiménez M.T., San Martín-Gonzalez M.F., Bello-Perez L.A. (2014). Protection of *L. rhamnosus* by spray-drying using two prebiotics colloids to enhance the viability. Carbohydr. Polym..

[222] dos Santos R.C.S., Finkler L., Finkler C.L.L. (2014). Microencapsulation of *Lactobacillus casei* by spray drying. J. Microencapsul..

[223] Rodrigues D., Sousa S., Rocha-Santos T., Silva J.P., Sousa Lobo J.M., Costa P., Amaral M.H., Pintado M.M., Gomes A.M., Malcata F.X. (2011). Influence of l-cysteine, oxygen and relative humidity upon survival throughout storage of probiotic bacteria in whey protein-based microcapsules. Int. Dairy J..

[224] Thantsha M.S., Guest J., Mputle I. (2011). Comparison of different methods for release of *Bifidobacterium longum* Bb46 from the poly(vinylpyrrolidone)-poly(vinylacetate-co-crotonic acid) interpolymer complex matrix, and the effect of grinding on the microparticles. World J. Microbiol. Biotechnol..

[225] De Prisco A., Maresca D., Ongeng D., Mauriello G. (2015). Microencapsulation by vibrating technology of the probiotic strain *Lactobacillus reuteri* DSM 17938 to enhance its survival in foods and in gastrointestinal environment. LWT Food Sci. Technol..

[226] Praepanitchai O.-A., Noomhorm A., Anal A.K. (2019). Survival and Behavior of Encapsulated Probiotics (*Lactobacillus plantarum*) in Calcium-Alginate-Soy Protein Isolate-Based Hydrogel Beads in Different Processing Conditions (pH and Temperature) and in Pasteurized Mango Juice. Biomed. Res. Int..

[227] Mortazavian A., Razavi S.H., Ehsani M.R., Sohrabvandi S. (2007). Principles and methods of microencapsulation of probiotic microorganisms. Iran. J. Biotechnol..

[228] Champagne C.P., Fustier P. (2007). Microencapsulation for the improved delivery of bioactive compounds into foods. Curr. Opin. Biotechnol..

[229] Behboudi-Jobbehdar S., Soukoulis C., Yonekura L., Fisk I. (2013). Optimization of Spray-Drying Process Conditions for the Production of Maximally Viable Microencapsulated *L. acidophilus* NCIMB 701748. Dry Technol..

[230] Fritzen-Freire C.B., Prudêncio E.S., Amboni R.D.M.C., Pinto S.S., Negrão-Murakami A.N., Murakami F.S. (2012). Microencapsulation of bifidobacteria by spray drying in the presence of prebiotics. Food Res. Int..

[231] Hernández-Rodríguez L., Lobato-Calleros C., Pimentel-González D.J., Vernon-Carter E.J. (2014). *Lactobacillus plantarum* protection by entrapment in whey protein isolate: κ-carrageenan complex coacervates. Food Hydrocoll..

[232] Ranadheera C.S., Evans C.A., Adams M.C., Baines S.K. (2015). Microencapsulation of *Lactobacillus acidophilus* LA-5, *Bifidobacterium animalis* subsp. *lactis* BB-12 and *Propionibacterium jensenii* 702 by spray drying in goat’s milk. Small Rumin. Res..

[233] Ying D., Schwander S., Weerakkody R., Sanguansri L., Gantenbein-Demarchi C., Augustin M.A. (2013). Microencapsulated *Lactobacillus rhamnosus* GG in whey protein and resistant starch matrices: Probiotic survival in fruit juice. J. Funct. Foods.

[234] Ivanovska T.P., Petrushevska-Tozi L., Grozdanov A., Petkovska R., Hadjieva J., Popovski E., Stafilov T., Mladenovska K. (2014). From optimization of synbiotic microparticles prepared by spray-drying to development of new functional carrot juice. Chem. Ind. Chem. Eng. Q..

[235] Nazzaro F., Fratianni F., Coppola R., Sada A., Orlando P. (2009). Fermentative ability of alginate-prebiotic encapsulated *Lactobacillus acidophilus* and survival under simulated gastrointestinal conditions. J. Funct. Foods.

[236] Amine K.M., Champagne C.P., Raymond Y., St-Gelais D., Britten M., Fustier P., Salmieri S., Lacroix M. (2014). Survival of microencapsulated *Bifidobacterium longum* in Cheddar cheese during production and storage. Food Control.

[237] Muthukumarasamy P., Holley R.A. (2006). Microbiological and sensory quality of dry fermented sausages containing alginate-microencapsulated *Lactobacillus reuteri*. Int. J. Food Microbiol..

[238] Rodrigues D., Sousa S., Gomes A., Pintado M., Silva J., Costa P., Amaral M., Rocha-Santos T., Freitas A. (2012). Storage Stability of *Lactobacillus paracasei* as Free Cells or Encapsulated in Alginate-Based Microcapsules in Low pH Fruit Juices. Food Bioprocess Technol..

[239] Doherty S.B., Auty M.A., Stanton C., Ross R.P., Fitzgerald G.F., Brodkorb A. (2012). Application of whey protein micro-bead coatings for enhanced strength and probiotic protection during fruit juice storage and gastric incubation. J. Microencapsul..

[240] Bhat A.R., Irorere V.U., Bartlett T., Hill D., Kedia G., Charalampopoulos D., Nualkaekul S., Radecka I. (2015). Improving survival of probiotic bacteria using bacterial poly-gamma-glutamic acid. Int. J. Food Microbiol..

[241] Nualkaekul S., Cook M.T., Khutoryanskiy V.V., Charalampopoulos D. (2013). Influence of encapsulation and coating materials on the survival of *Lactobacillus plantarum* and *Bifidobacterium longum* in fruit juices. Food Res. Int..

[242] Homayouni A., Azizi A., Ehsani M.R., Yarmand M.S., Razavi S.H. (2008). Effect of microencapsulation and resistant starch on the probiotic survival and sensory properties of synbiotic ice cream. Food Chem..

[243] Mortazavian A.M., Ehsani M.R., Azizi A., Razavi S.H., Mousavi S.M., Sohrabvandi S., Reinheimer J.A. (2008). Viability of calcium-alginate-microencapsulated probiotic bacteria in Iranian yogurt drink (Doogh) during refrigerated storage and under simulated gastrointestinal conditions. Aust. J. Dairy Technol..

[244] ÖZer B., Uzun Y.S., Kirmaci H.A. (2008). Effect of Microencapsulation on Viability of *Lactobacillus acidophilus* LA-5 and *Bifidobacterium bifidum* BB-12 During Kasar Cheese Ripening. Int. J. Dairy Technol..

[245] GonzÁLez-SÁNchez F., Azaola A., GutiÉRrez-LÓPez G.F., HernÁNdez-SÁNchez H. (2010). Viability of microencapsulated *Bifidobacterium animalis* ssp. *lactis* BB12 in kefir during refrigerated storage. Int. J. Dairy Technol..

[246] Ortakci F., Sert S. (2012). Stability of free and encapsulated *Lactobacillus acidophilus* ATCC 4356 in yogurt and in an artificial human gastric digestion system. J. Dairy Sci..

[247] Santillo A., Bevilacqua A., Corbo M.R., Sevi A., Sinigaglia M., Albenzio M. (2014). Functional Pecorino cheese production by using innovative lamb rennet paste. Innov. Food Sci. Emerg. Technol..

[248] Santillo A., Albenzio M., Bevilacqua A., Corbo M.R., Sevi A. (2012). Encapsulation of probiotic bacteria in lamb rennet paste: Effects on the quality of Pecorino cheese. J. Dairy Sci..

[249] Nualkaekul S., Lenton D., Cook M.T., Khutoryanskiy V.V., Charalampopoulos D. (2012). Chitosan coated alginate beads for the survival of microencapsulated Lactobacillus plantarum in pomegranate juice. Carbohydr. Polym..

[250] Özer B., Kirmaci H.A., Şenel E., Atamer M., Hayaloğlu A. (2009). Improving the viability of *Bifidobacterium bifidum* BB-12 and *Lactobacillus acidophilus* LA-5 in white-brined cheese by microencapsulation. Int. Dairy J..

[251] Ribeiro M.C.E., Chaves K.S., Gebara C., Infante F.N.S., Grosso C.R.F., Gigante M.L. (2014). Effect of microencapsulation of *Lactobacillus acidophilus* LA-5 on physicochemical, sensory and microbiological characteristics of stirred probiotic yoghurt. Food Res. Int..

[252] Mousa A., Liu X.M., Chen Y.Q., Zhang H., Chen W. (2014). Evaluation of physiochemical, textural, microbiological and sensory characteristics in set yogurt reinforced by microencapsulated *Bifidobacterium bifidum* F-35. Int. J. Food Sci. Technol..

[253] Ziar H., Gérard P., Riazi A. (2012). Calcium alginate-resistant starch mixed gel improved the survival of *Bifidobacterium animalis* subsp. *lactis* Bb12 and *Lactobacillus rhamnosus* LBRE-LSAS in yogurt and simulated gastrointestinal conditions. Int. J. Food Sci. Technol..

[254] Sandoval-Castilla O., Lobato-Calleros C., García-Galindo H.S., Alvarez-Ramírez J., Vernon-Carter E.J. (2010). Textural properties of alginate–pectin beads and survivability of entrapped *Lb. casei* in simulated gastrointestinal conditions and in yoghurt. Food Res. Int..

[255] Urbanska A.M., Bhathena J., Prakash S. (2007). Live encapsulated *Lactobacillus acidophilus* cells in yogurt for therapeutic oral delivery: Preparation and in vitro analysis of alginate–chitosan microcapsules. This article is one of a selection of papers published in this special issue (part 1 of 2) on the Safety and Efficacy of Natural Health Products. Can. J. Physiol. Pharm..

[256] Ahmadi A., Milani E., Madadlou A., Mortazavi S., Mokarram R., Salarbashi D. (2014). Synbiotic yogurt-ice cream produced via incorporation of microencapsulated *Lactobacillus acidophilus* (la-5) and fructooligosaccharide. J. Food Sci. Technol..

[257] Duenas M., Munoz-Gonzalez I., Cueva C., Jimenez-Giron A., Sanchez-Patan F., Santos-Buelga C., Moreno-Arribas M.V., Bartolome B. (2015). A survey of modulation of gut microbiota by dietary polyphenols. Biomed. Res. Int..

[258] Markowiak P., Śliżewska K. (2017). Effects of Probiotics, Prebiotics, and Synbiotics on Human Health. Nutrients.

[259] Ruiz-Moreno M.J., Muñoz-Redondo J.M., Cuevas F.J., Marrufo-Curtido A., León J.M., Ramírez P., Moreno-Rojas J.M. (2017). The influence of pre-fermentative maceration and ageing factors on ester profile and marker determination of Pedro Ximenez sparkling wines. Food Chem..

[260] Quigley E.M.M. (2019). Prebiotics and Probiotics in Digestive Health. Clin. Gastroenterol. Hepatol..

[261] Choque Delgado G.T., Tamashiro W. (2018). Role of prebiotics in regulation of microbiota and prevention of obesity. Food Res. Int..

[262] Kolida S., Gibson G.R. (2011). Synbiotics in health and disease. Annu. Rev. Food Sci. Technol..

[263] Asto E., Mendez I., Audivert S., Farran-Codina A., Espadaler J. (2019). The Efficacy of Probiotics, Prebiotic Inulin-Type Fructans, and Synbiotics in Human Ulcerative Colitis: A Systematic Review and Meta-Analysis. Nutrients.

[264] Notay M., Foolad N., Vaughn A.R., Sivamani R.K. (2017). Probiotics, Prebiotics, and Synbiotics for the Treatment and Prevention of Adult Dermatological Diseases. Am. J. Clin. Dermatol..

[265] Tian X., Pi Y.-P., Liu X.-L., Chen H., Chen W.-Q. (2018). Supplemented Use of Pre-, Pro-, and Synbiotics in Severe Acute Pancreatitis: An Updated Systematic Review and Meta-Analysis of 13 Randomized Controlled Trials. Front. Pharmacol..

[266] Mandal S., Hati S., Puniya A.K., Singh R., Singh K. (2013). Development of synbiotic milk chocolate using encapsulated *Lactobacillus casei* NCDC 298. J. Food Process Preserv..

[267] Di Criscio T., Fratianni A., Mignogna R., Cinquanta L., Coppola R., Sorrentino E., Panfili G. (2010). Production of functional probiotic, prebiotic, and synbiotic ice creams. J. Dairy Sci..

[268] Wu Y., Zhang G. (2018). Synbiotic encapsulation of probiotic *Latobacillus plantarum* by alginate-arabinoxylan composite microspheres. LWT Food Sci. Technol..

[269] Nakkarach A., Withayagiat U. (2018). Comparison of synbiotic beverages produced from riceberry malt extract using selected free and encapsulated probiotic lactic acid bacteria. Agric. Nat. Resour..

[270] Sathyabama S., Ranjith kumar M., Bruntha devi P., Vijayabharathi R., Brindha priyadharisini V. (2014). Co-encapsulation of probiotics with prebiotics on alginate matrix and its effect on viability in simulated gastric environment. LWT Food Sci. Technol..

[271] Atia A., Gomma A.I., Fliss I., Beyssac E., Garrait G., Subirade M. (2017). Molecular and biopharmaceutical investigation of alginate-inulin synbiotic coencapsulation of probiotic to target the colon. J. Microencapsul..

[272] Lerner A., Matthias T., Aminov R. (2017). Potential Effects of Horizontal Gene Exchange in the Human Gut. Front. Immunol..

